# Effective media properties of hyperuniform disordered composite materials

**DOI:** 10.1371/journal.pone.0185921

**Published:** 2017-10-05

**Authors:** Bi-Yi Wu, Xin-Qing Sheng, Yang Hao

**Affiliations:** 1 School of Electronic Engineering and Computer Science, Queen Mary University of London, London, United Kingdom; 2 Center for Electromagnetic Simulation, School of Information and Electronic Engineering, Beijing Institute of Technology, Beijing, People’s Republic of China; Beihang University, CHINA

## Abstract

The design challenge of new functional composite materials consisting of multiphase materials has attracted an increasing interest in recent years. In particular, understanding the role of distributions of ordered and disordered particles in a host media is scientifically and technologically important for designing novel materials and devices with superior spectral and angular properties. In this work, the effective medium property of disordered composite materials consisting of hyperuniformly distributed hard particles at different filling fractions is investigated. To accurately extract effective permittivity of a disordered composite material, a full-wave finite element method and the transmission line theory are used. Numerical results show that the theory of hyperuniformity can be conveniently used to design disordered composite materials with good accuracy compared with those materials with randomly dispersed particles. Furthermore, we demonstrate that a Luneburg lens based on the proposed hyperuniform media has superior radiation properties in comparison with previously reported metamaterial designs and it may open up a new avenue in electromagnetic materials-by-design.

## Introduction

Recently, research into disordered materials has grown immensely because of its ubiquity in natural and artificial systems [1, 2]. In optics, unlike conventional photonic crystals with regular lattice structures or quasicrystals, the hyperuniform disordered materials with statistical isotropy and constrained randomness had received increasing attention because of its large, complete photonic bandgaps for all directions and polarization. These distinct characteristics have also led to the development of a variety of novel devices including the free-form optical waveguide with arbitral bend angles [3], high-Q compact optical polarizer [4], on-chip spectrometers [5], and devices with low dielectric contrast but complete photonic band gap [6],etc.

Although different sets of disordered configurations with identical spatial Fourier spectra are statistically equivalent, the effective index of refraction *n*_*e*_ or effective permittivity and permeability of individual configurations are different. In various applications, it is critical to accurately predict electromagnetic properties of materials, such as gradient index media, tunable superlens using random composites [7]. Effective medium approaches such as the Maxwell-Garnett method [8], Bruggeman theory [9], Lichtennecker theory [10], etc. have been proposed to estimate the effective refraction index of composite materials using simple analytical approximations. They consider neither effects of shape, size, and arrangement of particle inclusions nor frequency and spatial dispersions. In photonic crystals, effective media properties are often obtained by calculating the band diagram from unit cells. The isofrequency contour in the band diagram directly reveals the information of spatial dispersion, yet the retrieval of effective refraction index is realized by finding a homogeneous media with same band diagram, which is proven to be very inefficient. This approach is limited to periodic structures and also commonly used in applications to metamaterials [7, 11]. For a general composite material, there are several homogenization techniques and they can be divided into two categories: the quasistatic approach is based on the long wavelength assumption [12–14], when, for example, the operating wavelength is much larger than the size of inclusion particles in composite materials; another approach homogenize composite materials by characterizing their electrodynamic responses based the transmission line theory [15–17] or the resonator approach [18, 19]. Corresponding numerical simulation methods include the plane wave expansion method [20] for periodic structures, the finite difference time domain (FDTD) method [15] and the multipole approximation method [21].

In this paper, we investigate effective media properties of hyperuniformly disordered materials and evaluate their performance in some potential electromagnetic applications. In particular, the effective medium of a two-phase composite consisting of hyperuniform infinitely long dielectric cylinders (for the two-dimensional case) is studied, since it represents a simple but general form of structural and kinetic properties of any matter [12]. Instead of using the quasi-static approximation, in this work, we extract the effective permittivity at a certain frequency from the reflection coefficient when the composite material is illuminated by a plane wave, and this process is rigorously modeled by the full-wave finite element method (FEM). We anticipate that the design approach can be used to “dial” the material for advanced electromagnetic applications in the future. The rest of this paper is organized as follows. Section II presents details of full wave numerical modeling and characterization of effective permittivities of proposed hyperuniform composite materials. Numerical results of several exemplary structures are summarized in section III. A gradient index media as Luneburg lens realized by hyperuniform composites and metamaterials are demonstrated and compared in section IV. Some conclusions and remarks are given in the last section.

## Homogenization of disordered composite materials

### Hyperuniform disordered materials

The concept of “hyperuniform” was first used to describe a point distribution pattern whose number variance *σ*(*R*) within a spherical sampling window of radius *R* increases at a rate slower than the window volume, i.e. slower than *R*^*d*^ where *d* is the number of dimensions [22]. In the Fourier space, hyperuniformity means that the structure factor *S*(**k**) approaches zero as |**k**| → 0. The formation of hyperuniformly disordered materials starts with the generation of hyperuniform point pattern, then it is generalized to structured particles, colloids, or bodies as is done for random media [23]. The hyperuniform point pattern is generated using so-called collective coordinate approach [24, 25], essentially it is a nonlinear optimization method to find a point pattern satisfying prescribed structure characteristics. The structure factor (*S*(**k**)) is proportional to the scattering intensity of an incident wave from a configuration of *N* particles **r**_1_, **r**_2_, …, **r**_*N*_ subjecting to periodic boundary condition and defined as

$$\begin{array}{c} S\left(k\right)=\frac{1}{N}|\sum_{j=1}^{N}\text{exp}\left(ik\cdot r_{j}\right){|}^{2} \end{array}$$ (1)

where **k** is the wave vector associated with the system and boundary conditions, e.g. in 2D case **k** = (2*πn*_*x*_/*L*_*x*_, 2*πn*_*y*_/*L*_*y*_), where $n_{x},n_{y}\in \mathbb{Z}$, *L*_*x*_, *L*_*y*_ are length of unit cell. We also use a constraining quantity related to structure factor as

$$\begin{array}{c} \begin{array}{cc} C(k) & =B\\ & =\sum_{i=1}^{N-1}\sum_{j=i+1}^{N}\text{cos}\left(k\cdot \left(r_{i}-r_{j}\right)\right) \end{array} \end{array}$$ (2)

This constraining quantity can be seen as an interaction pair potential between particle **r**_*i*_ and **r**_*j*_; then the total nonnegative potential energy can be written as

$$\begin{array}{c} \Phi =\Omega^{-1}\sum_{k}V\left(k\right)|C(k){|}^{2} \end{array}$$ (3)

where Ω is the system volume and *V*(**k**) is the auxiliary function. It is clearly that for any *V*(**k**) that is positive for |**k**| < *K* and zero otherwise, the minimum Φ in (3) would driving *C*(**k**) or *S*(**k**) to its minimum absolute value for all |**k**| < *K* [25]. For simplicity, we define the *V*(**k**) is constant *V*_0_ for all **k** ∈ **Q**, where **Q** is the set of wave vectors such that 0 < |**k**| < *K*, and all zero otherwise. Usually we find the minimum global potential energy corresponds to a particle distribution with *C*(**k**) = −*N*/2 for **k** ∈ **Q**. Generally, for designed structured factor *S*_0_(**k**) and its associated *C*_0_(**k**) by (2), the global potential energy satisfying

$$\begin{array}{c} \Phi =\Omega^{-1}\sum_{k}V\left(k\right)|C\left(k\right)-C_{0}\left(k\right){|}^{2} \end{array}$$ (4)

**Fig 1:**
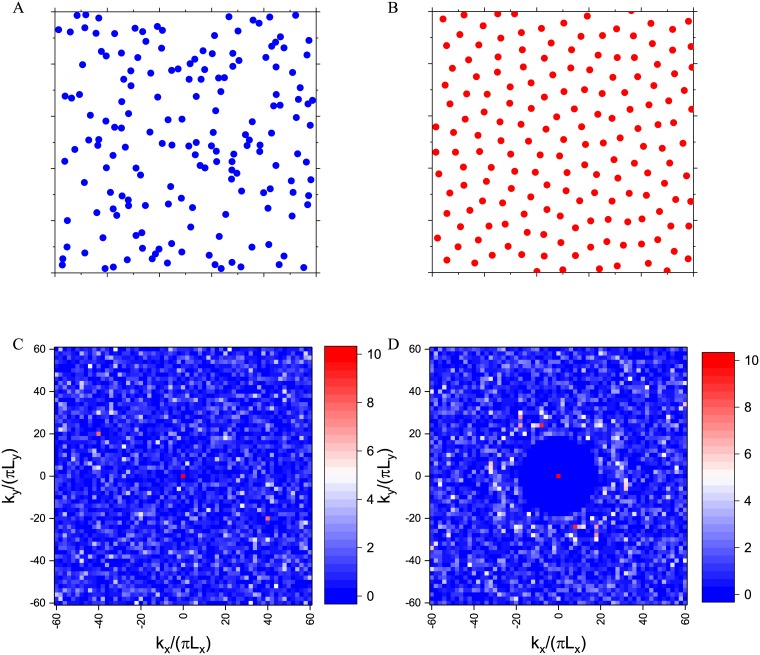
A random disordered point configuration (left panel) and hyperuniform disordered point configuration(right panel). It shows that it is difficult to detect the hyperuniformity from human eyes observation A and B, while the difference is quite clear in structure factors C and D.

To find the minimum value of global energy (3) or (4) and corresponding point pattern, MINOP [26] algorithm is commonly used [25]. Fig 1 presents two 2D disordered point configurations and their corresponding structure factors. From the point configuration, it is hard to detect the hyperunfromity by human eyes from these completely random point configurations, yet their structure factors are dramatically different. To describe the degree of the randomness in the point pattern, the constrain factor *χ* = *M*(*K*)/2*N* which is the ratio of constrained degrees of freedom to the total number of degrees of freedom is introduced in [25].

### Effective permittivity extraction

**Fig 2:**
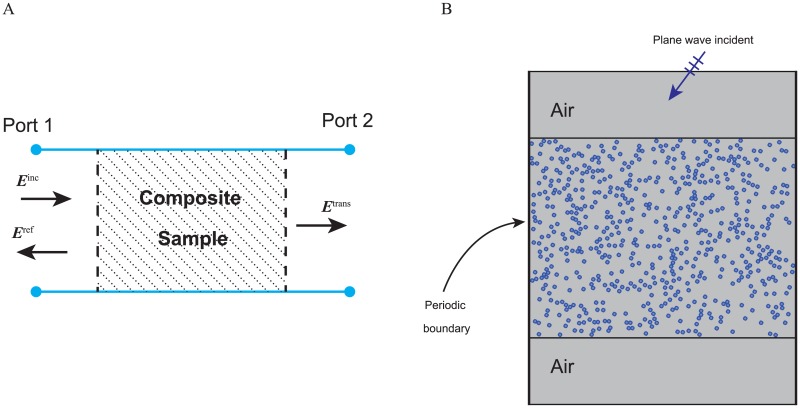
A schematic of Composite characterization using S-parameter retrieval. (A) A composite sample in transmission line. (B) Cross section view of composite material placed in layered medium.

In the last several decades, numerical simulation has proved itself a valuable tool for homogenization the dielectric properties of multiphase composites and their structures, which is very important because of the wide variety of heterostructures in nature. There are many methods to extract the effective dielectric property of a composite, such as S-parameter retrieval [15–17], resonator method [18, 19], plane wave expansion method [20], Clausius-Mossotti relation [21] and the field averaging technique [27]. Among these methods, the S-parameter retrieval method is accurate and versatile to use, because the S-parameters can be obtained either by simulation or measurement, while other methods mainly rely on the numerical simulation and computationally intensive. The S-parameter retrieval approach characterizes effective dielectric properties from the transmission and reflection coefficients of a composite loaded transmission line. Assuming the composite sample corresponds to one section of the transmission line as shown Fig 2A, and it has an effective relative permittivity *ϵ*_*e*_ and relative permeability *μ*_*e*_. Then the scattering parameters of the two-port transmission line are

$$\begin{array}{c} S_{11}=e^{-j2\beta_{0}L_{1}}\frac{\Gamma (1-z^{2})}{1-\Gamma^{2}z^{2}} \end{array}$$ (5)

$$\begin{array}{c} S_{22}=e^{-j2\beta_{0}L_{2}}\frac{\Gamma (1-z^{2})}{1-\Gamma^{2}z^{2}} \end{array}$$ (6)

$$\begin{array}{c} S_{12}=S_{21}=e^{-j\beta_{0}\left(L_{1}+L_{2}\right)}\frac{z(1-\Gamma^{2})}{1-\Gamma^{2}z^{2}} \end{array}$$ (7)

where *L*_1_ and *L*_2_ are the distances from sample to the transmission line terminal ports, and *β*_0_ is the propagation constant in empty transmission line *β*_0_ = *ω*/*c*_0_ with *ω* the angular frequency, *c*_0_ the speed of light in vacuum. Phase shift in composite sample is *z* = *e*^−*jβ*_*e*_*L*^ with the effective propagation constant $\beta_{e}=\sqrt{\epsilon_{e}\mu_{e}}\omega /c_{0}$ and the sample length *L*. The reflection coefficient from air to sample is

$$\begin{array}{c} \Gamma =\frac{Z_{s}-Z_{0}}{Z_{s}+Z_{0}} \end{array}$$ (8)

where *Z*_0_ and *Z*_*s*_ are wave impedance in air and homogenized composite. Specifically, for TE or TM waves, the reflection coefficients are

$$\begin{array}{c} \begin{array}{c} \Gamma =\frac{\beta_{0}/\mu_{0}-\beta_{e}/\mu_{e}}{\beta_{0}/\mu_{0}+\beta_{e}/\mu_{e}}\;\text{TE}\;\text{wave}\\ \Gamma =\frac{\beta_{e}/\epsilon_{e}-\beta_{0}/\epsilon_{0}}{\beta_{e}/\epsilon_{e}+\beta_{0}/\epsilon_{0}}\;\text{TM}\;\text{case} \end{array} \end{array}$$ (9)

Combing (5),(6) and (7) yields

$$\begin{array}{c} S_{12}S_{21}-S_{11}S_{22}=e^{-2j\beta_{0}\left(L_{1}+L_{2}\right)}\frac{z^{2}-\Gamma^{2}}{1-\Gamma^{2}z^{2}} \end{array}$$ (10)

then the effective parameter *ϵ*_*e*_ and *μ*_*e*_ can be found by solving this nonlinear equation. A more robust yet complicated version S-parameter retrieval method can be found in [17], which is designed to find the effective parameter for metamaterials near its resonance of a unit element. To find the S-parameters of a composite loaded transmission line, we prefer to use the FEM rather than other approaches. The FDTD method [15, 27] suffers from dispersion errors, and its staircase approximation cannot represent complex composite geometry accurately. The multipole approximation based on the Mie theory [21] limit to spherical inclusions. On the other hand, the FEM allows different types of mesh elements (i.e. triangular, tetrahedral or hexahedral elements), and it is more versatile in modeling complex geometries. In this study, we consider simulating the plane wave reflection from the composite samples as shown in Fig 2B, the composite sample is placed in a section of the transmission line, which scattering parameters can be obtained to reveal effective parameters of composite under test at its operating frequency. For simplicity, we validate this approach for two-dimensional cases, which the FEM simulation is reduced to solve the 2D Helmholtz equation. In this study, we use the widely used FEM solver COMSOL (electromagnetic wave frequency domain module) to find reflection and transmission coefficients of hyperuniform media under TE and TM illuminations.

## Numerical results

To demonstrate the validity of the proposed design approach, we have performed a number of calculations for composites with various hyperuniform and random configurations for different filling fractions. Here we present simulation results of effective permittivity of two-phase mixtures of hard particles and compare them with those obtained by using conventional analytical models.

First, we consider the effective permittivity for different filling fractions, in this study we consider three different kinds of composites: a triangular lattice 2D crystal, a hyperuniformly disordered and a randomly disordered composite. Namely, we fill a square primitive cell (length *L*) with a set of monodisperse cylindrical particles with (a) equilateral triangular (b) hyperuniform (c) random distribution. For low filling fractions, the random point distribution can be generated by using the random sequential addition (RSA) method [12]. This method generates random particle positions sequentially and accepts new random particle (satisfying uniform distribution) only if it is not overlapping with existing particles. As this acceptation and rejection process continues, it will become more time consuming to find a new region to place a new particle, and there is a saturation limit (for equal-sized circular particles, 55% filling fraction) above which no further addition is possible. For higher filling fractions, we generate random particle distributions using the molecular dynamic hard sphere packing method [28]. As the filling fraction goes higher, it imposes more constraints on particle distribution pattern, and the composite becomes wavy crystalline and then crystalline from disordered, thus in this study, the highest filling fraction for random composites is 80%.

The most popular effective theory for composite materials is the Maxwell-Garnett formula, the effective permittivity for two-phase composite is

$$\begin{array}{c} \frac{\epsilon_{\text{eff}}-\epsilon_{\text{host}}}{\epsilon_{\text{eff}}+\beta \epsilon_{\text{host}}}=f\frac{\epsilon_{\text{incl}}-\epsilon_{\text{host}}}{\epsilon_{\text{incl}}+\beta \epsilon_{\text{host}}} \end{array}$$ (11)

where *f* is the filling fraction of the inclusion material, *ϵ*_host_ and *ϵ*_incl_ are the relative permittivity of host and inclusion material respectively, here the factor *β* = 1 for two-dimensional composites with cylindrical inclusion and *β* = 2 for three-dimensional spherical particle inclusions. We note that for 2D composites consisting of isotropic particles, the effective permittivity in the direction of cylindrical inclusion axis is estimated by the linear formula *ϵ*_eff_ = (1−*f*)*ϵ*_host_ + *fϵ*_incl_.

**Fig 3:**
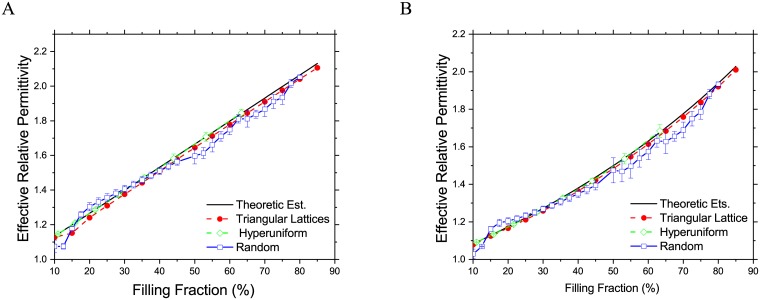
Effective relative permittivity of 2D composite materials with different filling fractions. (A) In the direction parallel to the inclusion cylinder axis. (B) In the transverse direction.

As shown in Fig 3, the effective relative permittivity of these three kinds of composites with different filling fractions at frequency λ/*d* = 20 are presented, where *d* is the diameter of cylinder inclusions and λ is the wavelength in vacuum. The host material is assumed as the vacuum, and the filling material dielectric constant is 2.33 at the operation frequency. The average effective permittivity for random and hyperuniform composites are obtained from 20 samples in this study. From this figure, we can see that the effective dielectric property of all these three kinds composites agrees well the theoretical estimation. However, the variance of random composites is much larger than that of hyperuniform materials. For large filling fractions (nearly 80%), there is less randomness in the point distribution, thus the variance also smaller. For hyperuniform composites, the hard particle filling fraction cannot be as high as that of random media configurations, in this work, the maximum filling fraction for 2D hyperuniform composite is about 60%.

**Fig 4:**
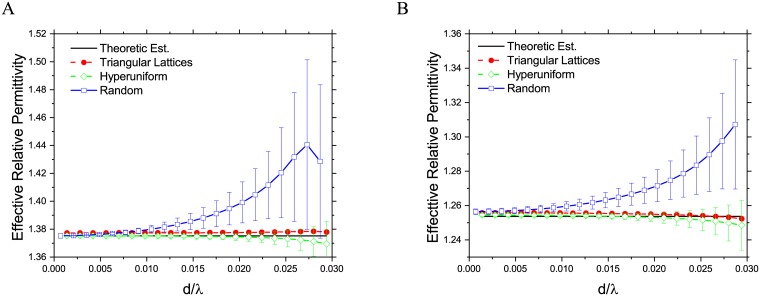
Effective relative permittivity of 2D composite materials under different frequency. (A) In the direction parallel to the inclusion cylinder axis. (B) In the transverse direction.

In previous studies, very few of them have studied frequency dispersion in the effective permittivity of composite materials. Under the long wavelength assumption, the effective permittivity of these three types composite at different frequencies is shown in Fig 4. In this frequency band, the dielectric property of both host and inclusion media have a constant relative permittivity (1.0 and 2.33 respectively), and the filling fractions in these composites are 28%. As shown in this figure, the variance of both random and hyperuniform composites increases as the operation frequency goes up, and yet again, the randomly dispersed composites have much larger variances comparing to hyperuniform configurations. The average effective permittivities of hyperuniform composites are also closer to that of the theoretical estimations. These results indicate that statistically, the random composite is more likely frequency dispersive than the hyperuniform counterpart. The effective dielectric properties of random composites are more susceptible to the inclusion particle positions comparing to hyperuniform disordered composites.

## A Luneburg lens from graded composites

In this section, we briefly report a design of lens with a graded dielectric to approximate the functionality of Luneburg lens which focuses a plane wave or transform the circular wave from a point source to plane wave. The dielectric constant of an ideal Luneburg lens is spatially dependent and the relative permittivity is a function of the lens’ radius *r* and written as

$$\begin{array}{c} \epsilon \left(r\right)=2-\frac{r^{2}}{R^{2}} \end{array}$$ (12)

where $r=\sqrt{x^{2}+y^{2}}$ is the distance to the lens center (assuming the 2D case in xy-plane) and *R* is the radius of the lens, the lens is magnetically inactive (i.e. *μ* = 1). The relative permittivity gradually decays from *ϵ* = 2 at the lens center to *ϵ* = 1 at the edge of the lens.

**Fig 5:**
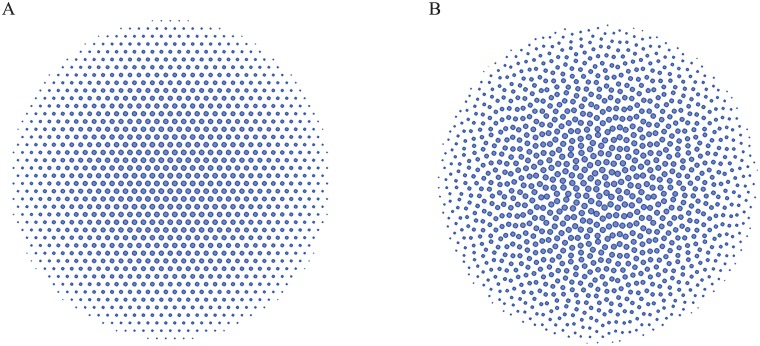
Two types of Luneburg lens designs. (A) Triangular lattice. (B) Hyperuniform point pattern.

Here we approximate the dielectric profile of Luneburg lens using 2D composites material with gradient refractive indices [29, 30], and two configurations are considered as shown in Fig 5. To generate these two designs, we first generate a triangular lattice (Fig 5A) and the same number points hyperuniform configuration (Fig 5B) in the square unit cell, then expand this cell in two dimensions periodically, and select those points located in the designed Luneburg lens. After finding the positions of the including fibers, the diameter of fibers is determined by required values of effective relative permittivity which is defined by (12) and the filling fraction obtained from the effective medium theory for electric field parallel to the fiber axis. In these two designs, the Luneburg lens has a diameter 0.6m, the edge length of unit cell *L* = 0.2*m*, the lattice distance for triangular lattice is *L*/12, thus there are 168 points in the unit cell. The inclusion material is glass fiber(SiO_2_ with dielectric constant 4.0), and their diameters vary from 1.10mm (at the lens rim) to 8.95mm (at the lens center). In the hyperuniform design, the constraint factor *χ* = 0.375, and the number of cylinder fibers in these two designs are 1174 for triangular lattice design and 1186 for hyperuniform design, respectively.

**Fig 6:**
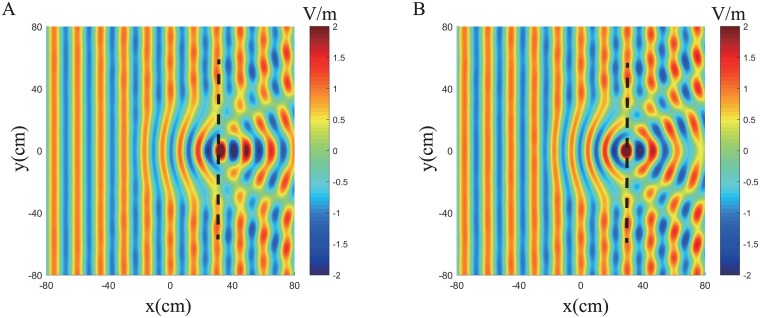
Electric field profile of two Luneburg designs under plane wave incidence from the left side at 2GHz. (A) the triangular lattice design. (B) The hyperuniform disordered design.

**Fig 7:**
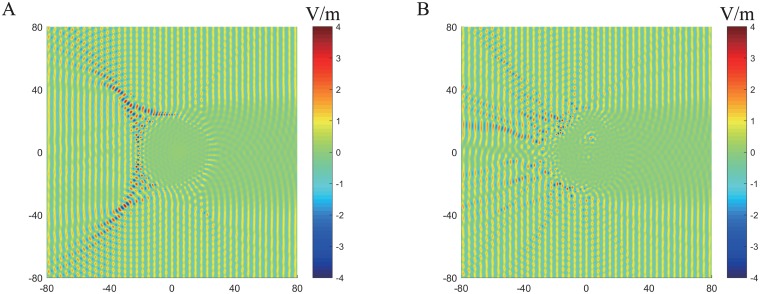
Electric field profile of two Luneburg designs under plane wave incidence from the left side at 8GHz. (A) the triangular lattice design. (B) The hyperuniform disordered design.

The electric field distribution for these two designs under plane wave incidence from the left are present in Figs 6 and 7 in which we can observe, in both designs, the structures convert the incident plane to the cylindrical wave perfectly at 2GHz. As the frequency increases, wave scattering in inhomogeneous composites become stronger and both designs will eventually lose lens functionality. At 8.0GHz, the metamaterial design reflects the incident wave along two specular directions due to the periodicity and rotational symmetry of particle inclusions, while the hyperuniform design scatters the incident wave randomly, resulting in diffused reflections.

**Fig 8:**
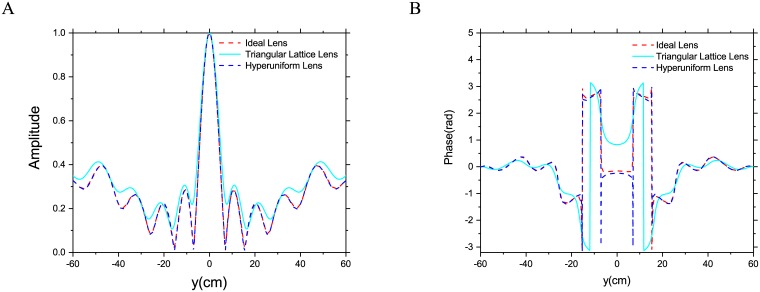
Normalized field intensity along the dash lines in Fig 6. (A) Amplitude. (B) Phase.

To further examine the performance of these two designs, the near field profile along the dashed line in Fig 6A and 6B are presented and compared with those of an ideal Luneburg lens in Fig 8. At 2GHz the normalized field intensity of the hyperuniform lens almost coincides with those of ideal lens, while the metamaterial lens demonstrates increased sidelobes and reduced broadside gain. The phase difference between these two designs is more obvious as shown in Fig 8B, the phase profile of hyperuniform lens also agree well those of ideal Luneburg lens. However, the phase profile of the triangular lattice lens is accurate only at the end of this observation line. The phase error between ideal Luneburg lens and hyperuniform lens near ±10° is because of the calculation error, the corresponding amplitude is nearly zero and this makes it difficult to calculate the phase angle accurately.

**Fig 9:**
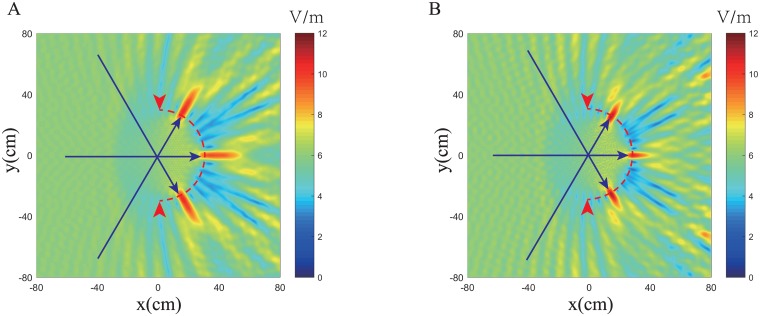
The electric field intensity of Luneburg lenses incident by three plane waves at incident angles of −60°,0° and 60°. (A) the triangular lattice design. (B) The hyperuniform disordered design.

**Fig 10:**
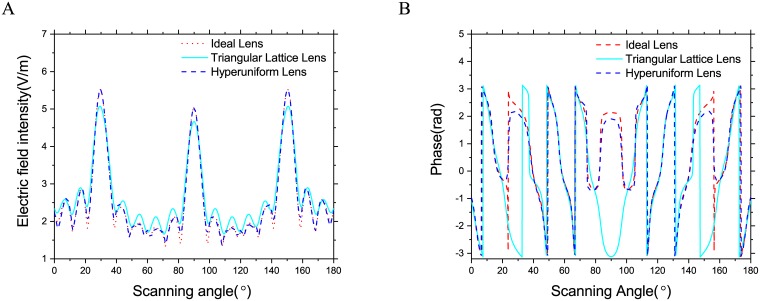
The electric field profile of focusing spots at the rim of the different Luneburg lenses shown in Fig 9 red dash lines. (A) Amplitude. (B) Phase.

To demonstrate the isotropy of the hyperuniform composite material, the electric field intensity distribution of the designed Luneburg lenses illuminated by three plane waves from different angles simultaneously at 3.0GHz is shown in Fig 9. Both two designs focus the incident wave to a spot at the opposite edge of the lens, yet the field distribution near the focusing spots of these two designs are slightly different. Thus, we calculate the field intensity profile at the rim of the lenses. The field intensity profiles, as well as those of ideal Luneburg lens, are presented in Fig 10. Again, the hyperuniform lens presents a better approximation to the ideal Luneburg lens comparing to the triangular lattice one. The intensity profile of the hyperuniform lens is symmetric and the peaks values are almost identical, and this indicates the hyperuniform composite is highly isotropic at this operating frequency. At 3GHz, the approximation error of both two designs become higher, nevertheless, the hyperuniform lens has better approximation accuracy comparing to the metamaterial counterpart. This result shows that the disordered hyperuniform design has a better focusing performance comparing to the metamaterial design and make it a better choice for the realization of next generation antennas based on transformation optics [31] such as the slim Luneburg lens [32], which has found ever-increasing engineering applications.

## Conclusion

Materials-by-design has been identified as a long-term vision for future innovations in science and engineering. In this work, it is verified that by applying hyperuniformity in the design of composites, we benefit from the subject of metamaterials whose material properties can be accurately predicted and the conventional composite material which is isotropic and homogeneous. Numerical results show that the effective permittivity of hyperuniform disordered composites has a much lower variance than those of random composite materials, and they agree very well with theoretical calculations. The frequency dispersion of the proposed hyperuniform composite is also lower than conventional random composites near the quasistatic limit. This indicates that the hyperuniform composite is a class of disordered composite with highly predictable material properties. We anticipate that with the help of emerging additive manufacturing technologies, such media can be found in many novel applications including Luneburg lens antennas as demonstrated in this paper.

## Supporting information

S1 File Configuration of triangular lattice lens. Positions (x,y-coordinates) and radius of each glass fiber in the triangular lattice Luneburg lens design.
(DOCX)

S2 File Configuration of hyperuniform lens. Positions (x,y-coordinates) and radius of each glass fiber in the hyperuniform Luneburg lens design.
(DOCX)

## References

[1] Wiersma DS. Disordered photonics. Nature Photonics. 2013;7(3):188–196. 10.1038/nphoton.2013.29

[2] Vynck K, Burresi M, Riboli F, Wiersma DS. Photon management in two-dimensional disordered media. Nature materials. 2012;11(12):1017–1022. 23042416 10.1038/nmat3442

[3] Man W, Florescu M, Williamson EP, He Y, Hashemizad SR, Leung BY, et al Isotropic band gaps and freeform waveguides observed in hyperuniform disordered photonic solids. Proceedings of the National Academy of Sciences. 2013;110(40):15886–15891. 10.1073/pnas.1307879110 PMC3791749 24043795

[4] Zhou W, Cheng Z, Zhu B, Sun X, Tsang HK. Hyperuniform Disordered Network Polarizers. IEEE Journal of Selected Topics in Quantum Electronics. 2016;22(6):288–294. 10.1109/JSTQE.2016.2528125

[5] Redding B, Liew SF, Sarma R, Cao H. Compact spectrometer based on a disordered photonic chip. Nature Photonics. 2013;7(9):746–751. 10.1038/nphoton.2013.190

[6] Man W, Florescu M, Matsuyama K, Yadak P, Nahal G, Hashemizad S, et al Photonic band gap in isotropic hyperuniform disordered solids with low dielectric contrast. Optics express. 2013;21(17):19972–19981. 10.1364/OE.21.019972 24105543

[7] Cai W, M SV. Optical Metamaterials:Fundalmentals and applications. NewYork:Springer; 2010.

[8] Garnett J. Colors in material glasses and metal films. Trans Roy Soc. 1904;53:385 10.1098/rsta.1904.0024

[9] Bruggeman VD. Berechnung verschiedener physikalischer Konstanten von heterogenen Substanzen. I. Dielektrizitätskonstanten und Leitfähigkeiten der Mischkörper aus isotropen Substanzen. Annalen der physik. 1935;416(7):636–664. 10.1002/andp.19354160705

[10] Lichtenecker K, Rother K. Die Herleitung des logarithmischen Mischungsgesetzes aus allgemeinen Prinzipien der stationären Strömung. phys Z. 1931;32:255–260.

[11] Hao Y, Mittra R. FDTD modeling of metamaterials: Theory and applications. Artech house; 2008.

[12] Myroshnychenko V, Brosseau C. Finite-element method for calculation of the effective permittivity of random inhomogeneous media. Physical Review E. 2005;71(1):016701 10.1103/PhysRevE.71.016701 15697758

[13] Tuncer E, Gubański SM, Nettelblad B. Dielectric relaxation in dielectric mixtures: Application of the finite element method and its comparison with dielectric mixture formulas. Journal of Applied Physics. 2001;89(12):8092–8100. 10.1063/1.1372363

[14] Koledintseva MY, Patil SK, Schwartz RW, Huebner W, Rozanov KN, Shen J, et al Prediction of effective permittivity of diphasic dielectrics as a function of frequency. IEEE Transactions on Dielectrics and Electrical Insulation. 2009;16(3):793–808. 10.1109/TDEI.2009.5128520

[15] Karkkainen KK, Sihvola AH, Nikoskinen KI. Effective permittivity of mixtures: numerical validation by the FDTD method. IEEE Transactions on Geoscience and Remote Sensing. 2000;38(3):1303–1308. 10.1109/36.843023

[16] Baker-Jarvis J, Vanzura EJ, Kissick WA. Improved technique for determining complex permittivity with the transmission/reflection method. IEEE Transactions on Microwave Theory and Techniques. 1990;38(8):1096–1103. 10.1109/22.57336

[17] Chen X, Grzegorczyk TM, Wu BI, Pacheco J Jr, Kong JA. Robust method to retrieve the constitutive effective parameters of metamaterials. Physical Review E. 2004;70(1):016608 10.1103/PhysRevE.70.016608 15324190

[18] Hakki B, Coleman P. A dielectric resonator method of measuring inductive capacities in the millimeter range. IRE Transactions on Microwave Theory and Techniques. 1960;8(4):402–410. 10.1109/TMTT.1960.1124749

[19] Bernard P, Gautray J. Measurement of dielectric constant using a microstrip ring resonator. IEEE Transactions on Microwave Theory and Techniques. 1991;39(3):592–595. 10.1109/22.75310

[20] Volkov S, Saarinen JJ, Sipe J. Effective medium theory for 2D disordered structures: a comparison to numerical simulations. Journal of Modern Optics. 2012;59(11):954–961. 10.1080/09500340.2012.685187

[21] Malasi A, Kalyanaraman R, Garcia H. From Mie to Fresnel through effective medium approximation with multipole contributions. Journal of Optics. 2014;16(6):065001 10.1088/2040-8978/16/6/065001

[22] Torquato S, Stillinger FH. Local density fluctuations, hyperuniformity, and order metrics. Physical Review E. 2003;68(4):041113 10.1103/PhysRevE.68.041113 14682929

[23] Torquato S. Random heterogeneous materials: microstructure and macroscopic properties. vol. 16 Springer Science & Business Media; 2013.

[24] Torquato S, Zhang G, Stillinger F. Ensemble theory for stealthy hyperuniform disordered ground states. Physical Review X. 2015;5(2):021020 10.1103/PhysRevX.5.021020

[25] Uche OU, Stillinger FH, Torquato S. Constraints on collective density variables: Two dimensions. Physical Review E. 2004;70(4):046122 10.1103/PhysRevE.70.046122 15600475

[26] Uche OU, Torquato S, Stillinger FH. Collective coordinate control of density distributions. Physical Review E. 2006;74(3):031104 10.1103/PhysRevE.74.031104 17025591

[27] Wu D, Chen J, Liu C. Numerical evaluation of effective dielectric properties of three-dimensional composite materials with arbitrary inclusions using a finite-difference time-domain method. Journal of applied physics. 2007;102(2):024107 10.1063/1.2756089

[28] Skoge M, Donev A, Stillinger FH, Torquato S. Packing hyperspheres in high-dimensional Euclidean spaces. Physical Review E. 2006;74(4):041127 10.1103/PhysRevE.74.041127 17155042

[29] Dyachenko PN, Pavelyev VS, Soifer VA. Graded photonic quasicrystals. Optics letters. 2012;37(12):2178–2180. 10.1364/OL.37.002178 22739847

[30] Bao D, Rajab KZ, Hao Y, Kallos E, Tang W, Argyropoulos C, et al All-dielectric invisibility cloaks made of BaTiO3-loaded polyurethane foam. New Journal of Physics. 2011;13(10):103023 10.1088/1367-2630/13/10/103023

[31] Quevedo-Teruel O, Tang W, Mitchell-Thomas RC, Dyke A, Dyke H, Zhang L, et al Transformation optics for antennas: why limit the bandwidth with metamaterials? Scientific reports. 2013;3:1903 10.1038/srep01903 23712699 PMC3664901

[32] Demetriadou A, Hao Y. Slim Luneburg lens for antenna applications. Optics express. 2011;19(21):19925–19934. 10.1364/OE.19.019925 21997001

