# Supplementary material for: Effective media properties of hyperuniform disordered composite materials
Source: PLoS One. 2017 Oct 5;12(10):e0185921. doi: 10.1371/journal.pone.0185921 (PMC5628886; doi:10.1371/journal.pone.0185921)
Supplement: S1 File — Positions (x,y-coordinates) and radius of each glass fiber in the triangular lattice Luneburg lens design. (DOCX) [file pone.0185921.s001.docx]

S1File

Glass fiber configurations of triangular lattice Luneburg lens design.

| X-coordinate(cm) | Y-coordinate(cm) | Radius(cm) |
| --- | --- | --- |
| -10 | -28.0385 | 0.05553 |
| -8.33333 | -28.0385 | 0.099447 |
| -6.66667 | -28.0385 | 0.124331 |
| -5 | -28.0385 | 0.140675 |
| -3.33333 | -28.0385 | 0.151271 |
| -1.66667 | -28.0385 | 0.157287 |
| 0 | -28.0385 | 0.159242 |
| 1.666667 | -28.0385 | 0.157287 |
| 3.333333 | -28.0385 | 0.151271 |
| 5 | -28.0385 | 0.140675 |
| 6.666667 | -28.0385 | 0.124331 |
| 8.333333 | -28.0385 | 0.099447 |
| 10 | -28.0385 | 0.05553 |
| -15 | -25.1517 | 0.097171 |
| -13.3333 | -25.1517 | 0.141282 |
| -11.6667 | -25.1517 | 0.171002 |
| -10 | -25.1517 | 0.193094 |
| -8.33333 | -25.1517 | 0.209979 |
| -6.66667 | -25.1517 | 0.222845 |
| -5 | -25.1517 | 0.232359 |
| -3.33333 | -25.1517 | 0.238924 |
| -1.66667 | -25.1517 | 0.242777 |
| 0 | -25.1517 | 0.244048 |
| 1.666667 | -25.1517 | 0.242777 |
| 3.333333 | -25.1517 | 0.238924 |
| 5 | -25.1517 | 0.232359 |
| 6.666667 | -25.1517 | 0.222845 |
| 8.333333 | -25.1517 | 0.209979 |
| 10 | -25.1517 | 0.193094 |
| 11.66667 | -25.1517 | 0.171002 |
| 13.33333 | -25.1517 | 0.141282 |
| 15 | -25.1517 | 0.097171 |
| -20 | -22.265 | 0.030844 |
| -18.3333 | -22.265 | 0.123216 |
| -16.6667 | -22.265 | 0.167856 |
| -15 | -22.265 | 0.199828 |
| -13.3333 | -22.265 | 0.22461 |
| -11.6667 | -22.265 | 0.244399 |
| -10 | -22.265 | 0.260335 |
| -8.33333 | -22.265 | 0.273094 |
| -6.66667 | -22.265 | 0.283106 |
| -5 | -22.265 | 0.290654 |
| -3.33333 | -22.265 | 0.295928 |
| -1.66667 | -22.265 | 0.299048 |
| 0 | -22.265 | 0.300081 |
| 1.666667 | -22.265 | 0.299048 |
| 3.333333 | -22.265 | 0.295928 |
| 5 | -22.265 | 0.290654 |
| 6.666667 | -22.265 | 0.283106 |
| 8.333333 | -22.265 | 0.273094 |
| 10 | -22.265 | 0.260335 |
| 11.66667 | -22.265 | 0.244399 |
| 13.33333 | -22.265 | 0.22461 |
| 15 | -22.265 | 0.199828 |
| 16.66667 | -22.265 | 0.167856 |
| 18.33333 | -22.265 | 0.123216 |
| 20 | -22.265 | 0.030844 |
| -21.6667 | -19.3782 | 0.110724 |
| -20 | -19.3782 | 0.166517 |
| -18.3333 | -19.3782 | 0.204839 |
| -16.6667 | -19.3782 | 0.234419 |
| -15 | -19.3782 | 0.258279 |
| -13.3333 | -19.3782 | 0.277897 |
| -11.6667 | -19.3782 | 0.294122 |
| -10 | -19.3782 | 0.307492 |
| -8.33333 | -19.3782 | 0.318366 |
| -6.66667 | -19.3782 | 0.326995 |
| -5 | -19.3782 | 0.333552 |
| -3.33333 | -19.3782 | 0.338157 |
| -1.66667 | -19.3782 | 0.340891 |
| 0 | -19.3782 | 0.341797 |
| 1.666667 | -19.3782 | 0.340891 |
| 3.333333 | -19.3782 | 0.338157 |
| 5 | -19.3782 | 0.333552 |
| 6.666667 | -19.3782 | 0.326995 |
| 8.333333 | -19.3782 | 0.318366 |
| 10 | -19.3782 | 0.307492 |
| 11.66667 | -19.3782 | 0.294122 |
| 13.33333 | -19.3782 | 0.277897 |
| 15 | -19.3782 | 0.258279 |
| 16.66667 | -19.3782 | 0.234419 |
| 18.33333 | -19.3782 | 0.204839 |
| 20 | -19.3782 | 0.166517 |
| 21.66667 | -19.3782 | 0.110724 |
| -25 | -16.4915 | 0.025985 |
| -23.3333 | -16.4915 | 0.136449 |
| -21.6667 | -16.4915 | 0.187947 |
| -20 | -16.4915 | 0.225372 |
| -18.3333 | -16.4915 | 0.254997 |
| -16.6667 | -16.4915 | 0.279315 |
| -15 | -16.4915 | 0.299621 |
| -13.3333 | -16.4915 | 0.316687 |
| -11.6667 | -16.4915 | 0.331016 |
| -10 | -16.4915 | 0.342951 |
| -8.33333 | -16.4915 | 0.352734 |
| -6.66667 | -16.4915 | 0.360541 |
| -5 | -16.4915 | 0.366498 |
| -3.33333 | -16.4915 | 0.370695 |
| -1.66667 | -16.4915 | 0.37319 |
| 0 | -16.4915 | 0.374018 |
| 1.666667 | -16.4915 | 0.37319 |
| 3.333333 | -16.4915 | 0.370695 |
| 5 | -16.4915 | 0.366498 |
| 6.666667 | -16.4915 | 0.360541 |
| 8.333333 | -16.4915 | 0.352734 |
| 10 | -16.4915 | 0.342951 |
| 11.66667 | -16.4915 | 0.331016 |
| 13.33333 | -16.4915 | 0.316687 |
| 15 | -16.4915 | 0.299621 |
| 16.66667 | -16.4915 | 0.279315 |
| 18.33333 | -16.4915 | 0.254997 |
| 20 | -16.4915 | 0.225372 |
| 21.66667 | -16.4915 | 0.187947 |
| 23.33333 | -16.4915 | 0.136449 |
| 25 | -16.4915 | 0.025985 |
| -26.6667 | -13.6047 | 0.029095 |
| -25 | -13.6047 | 0.141517 |
| -23.3333 | -13.6047 | 0.19486 |
| -21.6667 | -13.6047 | 0.233829 |
| -20 | -13.6047 | 0.264848 |
| -18.3333 | -13.6047 | 0.290474 |
| -16.6667 | -13.6047 | 0.312039 |
| -15 | -13.6047 | 0.33034 |
| -13.3333 | -13.6047 | 0.345894 |
| -11.6667 | -13.6047 | 0.35906 |
| -10 | -13.6047 | 0.370091 |
| -8.33333 | -13.6047 | 0.379175 |
| -6.66667 | -13.6047 | 0.386448 |
| -5 | -13.6047 | 0.392012 |
| -3.33333 | -13.6047 | 0.395938 |
| -1.66667 | -13.6047 | 0.398275 |
| 0 | -13.6047 | 0.399051 |
| 1.666667 | -13.6047 | 0.398275 |
| 3.333333 | -13.6047 | 0.395938 |
| 5 | -13.6047 | 0.392012 |
| 6.666667 | -13.6047 | 0.386448 |
| 8.333333 | -13.6047 | 0.379175 |
| 10 | -13.6047 | 0.370091 |
| 11.66667 | -13.6047 | 0.35906 |
| 13.33333 | -13.6047 | 0.345894 |
| 15 | -13.6047 | 0.33034 |
| 16.66667 | -13.6047 | 0.312039 |
| 18.33333 | -13.6047 | 0.290474 |
| 20 | -13.6047 | 0.264848 |
| 21.66667 | -13.6047 | 0.233829 |
| 23.33333 | -13.6047 | 0.19486 |
| 25 | -13.6047 | 0.141517 |
| 26.66667 | -13.6047 | 0.029095 |
| -26.6667 | -10.718 | 0.128398 |
| -25 | -10.718 | 0.188857 |
| -23.3333 | -10.718 | 0.231538 |
| -21.6667 | -10.718 | 0.265171 |
| -20 | -10.718 | 0.292889 |
| -18.3333 | -10.718 | 0.316251 |
| -16.6667 | -10.718 | 0.336167 |
| -15 | -10.718 | 0.35322 |
| -13.3333 | -10.718 | 0.367808 |
| -11.6667 | -10.718 | 0.380215 |
| -10 | -10.718 | 0.390649 |
| -8.33333 | -10.718 | 0.399266 |
| -6.66667 | -10.718 | 0.406179 |
| -5 | -10.718 | 0.411476 |
| -3.33333 | -10.718 | 0.415218 |
| -1.66667 | -10.718 | 0.417448 |
| 0 | -10.718 | 0.418188 |
| 1.666667 | -10.718 | 0.417448 |
| 3.333333 | -10.718 | 0.415218 |
| 5 | -10.718 | 0.411476 |
| 6.666667 | -10.718 | 0.406179 |
| 8.333333 | -10.718 | 0.399266 |
| 10 | -10.718 | 0.390649 |
| 11.66667 | -10.718 | 0.380215 |
| 13.33333 | -10.718 | 0.367808 |
| 15 | -10.718 | 0.35322 |
| 16.66667 | -10.718 | 0.336167 |
| 18.33333 | -10.718 | 0.316251 |
| 20 | -10.718 | 0.292889 |
| 21.66667 | -10.718 | 0.265171 |
| 23.33333 | -10.718 | 0.231538 |
| 25 | -10.718 | 0.188857 |
| 26.66667 | -10.718 | 0.128398 |
| -28.3333 | -7.83122 | 0.089416 |
| -26.6667 | -7.83122 | 0.168563 |
| -25 | -7.83122 | 0.21816 |
| -23.3333 | -7.83122 | 0.256002 |
| -21.6667 | -7.83122 | 0.28678 |
| -20 | -7.83122 | 0.312588 |
| -18.3333 | -7.83122 | 0.334577 |
| -16.6667 | -7.83122 | 0.353462 |
| -15 | -7.83122 | 0.369718 |
| -13.3333 | -7.83122 | 0.383679 |
| -11.6667 | -7.83122 | 0.395589 |
| -10 | -7.83122 | 0.405628 |
| -8.33333 | -7.83122 | 0.413933 |
| -6.66667 | -7.83122 | 0.420605 |
| -5 | -7.83122 | 0.425723 |
| -3.33333 | -7.83122 | 0.429341 |
| -1.66667 | -7.83122 | 0.431497 |
| 0 | -7.83122 | 0.432213 |
| 1.666667 | -7.83122 | 0.431497 |
| 3.333333 | -7.83122 | 0.429341 |
| 5 | -7.83122 | 0.425723 |
| 6.666667 | -7.83122 | 0.420605 |
| 8.333333 | -7.83122 | 0.413933 |
| 10 | -7.83122 | 0.405628 |
| 11.66667 | -7.83122 | 0.395589 |
| 13.33333 | -7.83122 | 0.383679 |
| 15 | -7.83122 | 0.369718 |
| 16.66667 | -7.83122 | 0.353462 |
| 18.33333 | -7.83122 | 0.334577 |
| 20 | -7.83122 | 0.312588 |
| 21.66667 | -7.83122 | 0.28678 |
| 23.33333 | -7.83122 | 0.256002 |
| 25 | -7.83122 | 0.21816 |
| 26.66667 | -7.83122 | 0.168563 |
| 28.33333 | -7.83122 | 0.089416 |
| -28.3333 | -4.94447 | 0.127319 |
| -26.6667 | -4.94447 | 0.191385 |
| -25 | -4.94447 | 0.236239 |
| -23.3333 | -4.94447 | 0.271573 |
| -21.6667 | -4.94447 | 0.300762 |
| -20 | -4.94447 | 0.325463 |
| -18.3333 | -4.94447 | 0.346636 |
| -16.6667 | -4.94447 | 0.364897 |
| -15 | -4.94447 | 0.380665 |
| -13.3333 | -4.94447 | 0.394239 |
| -11.6667 | -4.94447 | 0.405839 |
| -10 | -4.94447 | 0.415631 |
| -8.33333 | -4.94447 | 0.423739 |
| -6.66667 | -4.94447 | 0.43026 |
| -5 | -4.94447 | 0.435264 |
| -3.33333 | -4.94447 | 0.438803 |
| -1.66667 | -4.94447 | 0.440913 |
| 0 | -4.94447 | 0.441614 |
| 1.666667 | -4.94447 | 0.440913 |
| 3.333333 | -4.94447 | 0.438803 |
| 5 | -4.94447 | 0.435264 |
| 6.666667 | -4.94447 | 0.43026 |
| 8.333333 | -4.94447 | 0.423739 |
| 10 | -4.94447 | 0.415631 |
| 11.66667 | -4.94447 | 0.405839 |
| 13.33333 | -4.94447 | 0.394239 |
| 15 | -4.94447 | 0.380665 |
| 16.66667 | -4.94447 | 0.364897 |
| 18.33333 | -4.94447 | 0.346636 |
| 20 | -4.94447 | 0.325463 |
| 21.66667 | -4.94447 | 0.300762 |
| 23.33333 | -4.94447 | 0.271573 |
| 25 | -4.94447 | 0.236239 |
| 26.66667 | -4.94447 | 0.191385 |
| 28.33333 | -4.94447 | 0.127319 |
| -28.3333 | -2.05771 | 0.143918 |
| -26.6667 | -2.05771 | 0.202807 |
| -25 | -2.05771 | 0.245583 |
| -23.3333 | -2.05771 | 0.27974 |
| -21.6667 | -2.05771 | 0.308156 |
| -20 | -2.05771 | 0.332308 |
| -18.3333 | -2.05771 | 0.353071 |
| -16.6667 | -2.05771 | 0.371016 |
| -15 | -2.05771 | 0.386534 |
| -13.3333 | -2.05771 | 0.399909 |
| -11.6667 | -2.05771 | 0.411349 |
| -10 | -2.05771 | 0.421012 |
| -8.33333 | -2.05771 | 0.429019 |
| -6.66667 | -2.05771 | 0.435461 |
| -5 | -2.05771 | 0.440406 |
| -3.33333 | -2.05771 | 0.443904 |
| -1.66667 | -2.05771 | 0.44599 |
| 0 | -2.05771 | 0.446683 |
| 1.666667 | -2.05771 | 0.44599 |
| 3.333333 | -2.05771 | 0.443904 |
| 5 | -2.05771 | 0.440406 |
| 6.666667 | -2.05771 | 0.435461 |
| 8.333333 | -2.05771 | 0.429019 |
| 10 | -2.05771 | 0.421012 |
| 11.66667 | -2.05771 | 0.411349 |
| 13.33333 | -2.05771 | 0.399909 |
| 15 | -2.05771 | 0.386534 |
| 16.66667 | -2.05771 | 0.371016 |
| 18.33333 | -2.05771 | 0.353071 |
| 20 | -2.05771 | 0.332308 |
| 21.66667 | -2.05771 | 0.308156 |
| 23.33333 | -2.05771 | 0.27974 |
| 25 | -2.05771 | 0.245583 |
| 26.66667 | -2.05771 | 0.202807 |
| 28.33333 | -2.05771 | 0.143918 |
| -28.3333 | 0.829038 | 0.146637 |
| -26.6667 | 0.829038 | 0.204745 |
| -25 | 0.829038 | 0.247187 |
| -23.3333 | 0.829038 | 0.281148 |
| -21.6667 | 0.829038 | 0.309435 |
| -20 | 0.829038 | 0.333494 |
| -18.3333 | 0.829038 | 0.354188 |
| -16.6667 | 0.829038 | 0.372079 |
| -15 | 0.829038 | 0.387555 |
| -13.3333 | 0.829038 | 0.400895 |
| -11.6667 | 0.829038 | 0.412308 |
| -10 | 0.829038 | 0.42195 |
| -8.33333 | 0.829038 | 0.429939 |
| -6.66667 | 0.829038 | 0.436367 |
| -5 | 0.829038 | 0.441302 |
| -3.33333 | 0.829038 | 0.444793 |
| -1.66667 | 0.829038 | 0.446875 |
| 0 | 0.829038 | 0.447566 |
| 1.666667 | 0.829038 | 0.446875 |
| 3.333333 | 0.829038 | 0.444793 |
| 5 | 0.829038 | 0.441302 |
| 6.666667 | 0.829038 | 0.436367 |
| 8.333333 | 0.829038 | 0.429939 |
| 10 | 0.829038 | 0.42195 |
| 11.66667 | 0.829038 | 0.412308 |
| 13.33333 | 0.829038 | 0.400895 |
| 15 | 0.829038 | 0.387555 |
| 16.66667 | 0.829038 | 0.372079 |
| 18.33333 | 0.829038 | 0.354188 |
| 20 | 0.829038 | 0.333494 |
| 21.66667 | 0.829038 | 0.309435 |
| 23.33333 | 0.829038 | 0.281148 |
| 25 | 0.829038 | 0.247187 |
| 26.66667 | 0.829038 | 0.204745 |
| 28.33333 | 0.829038 | 0.146637 |
| -28.3333 | 3.715789 | 0.136309 |
| -26.6667 | 3.715789 | 0.19748 |
| -25 | 3.715789 | 0.241203 |
| -23.3333 | 3.715789 | 0.275902 |
| -21.6667 | 3.715789 | 0.304677 |
| -20 | 3.715789 | 0.329084 |
| -18.3333 | 3.715789 | 0.350039 |
| -16.6667 | 3.715789 | 0.368131 |
| -15 | 3.715789 | 0.383766 |
| -13.3333 | 3.715789 | 0.397234 |
| -11.6667 | 3.715789 | 0.408749 |
| -10 | 3.715789 | 0.418472 |
| -8.33333 | 3.715789 | 0.426527 |
| -6.66667 | 3.715789 | 0.433005 |
| -5 | 3.715789 | 0.437978 |
| -3.33333 | 3.715789 | 0.441496 |
| -1.66667 | 3.715789 | 0.443593 |
| 0 | 3.715789 | 0.44429 |
| 1.666667 | 3.715789 | 0.443593 |
| 3.333333 | 3.715789 | 0.441496 |
| 5 | 3.715789 | 0.437978 |
| 6.666667 | 3.715789 | 0.433005 |
| 8.333333 | 3.715789 | 0.426527 |
| 10 | 3.715789 | 0.418472 |
| 11.66667 | 3.715789 | 0.408749 |
| 13.33333 | 3.715789 | 0.397234 |
| 15 | 3.715789 | 0.383766 |
| 16.66667 | 3.715789 | 0.368131 |
| 18.33333 | 3.715789 | 0.350039 |
| 20 | 3.715789 | 0.329084 |
| 21.66667 | 3.715789 | 0.304677 |
| 23.33333 | 3.715789 | 0.275902 |
| 25 | 3.715789 | 0.241203 |
| 26.66667 | 3.715789 | 0.19748 |
| 28.33333 | 3.715789 | 0.136309 |
| -28.3333 | 6.60254 | 0.109295 |
| -26.6667 | 6.60254 | 0.179899 |
| -25 | 6.60254 | 0.227033 |
| -23.3333 | 6.60254 | 0.263605 |
| -21.6667 | 6.60254 | 0.293587 |
| -20 | 6.60254 | 0.318844 |
| -18.3333 | 6.60254 | 0.340429 |
| -16.6667 | 6.60254 | 0.359006 |
| -15 | 6.60254 | 0.375022 |
| -13.3333 | 6.60254 | 0.388793 |
| -11.6667 | 6.60254 | 0.400551 |
| -10 | 6.60254 | 0.410468 |
| -8.33333 | 6.60254 | 0.418677 |
| -6.66667 | 6.60254 | 0.425275 |
| -5 | 6.60254 | 0.430337 |
| -3.33333 | 6.60254 | 0.433917 |
| -1.66667 | 6.60254 | 0.43605 |
| 0 | 6.60254 | 0.436759 |
| 1.666667 | 6.60254 | 0.43605 |
| 3.333333 | 6.60254 | 0.433917 |
| 5 | 6.60254 | 0.430337 |
| 6.666667 | 6.60254 | 0.425275 |
| 8.333333 | 6.60254 | 0.418677 |
| 10 | 6.60254 | 0.410468 |
| 11.66667 | 6.60254 | 0.400551 |
| 13.33333 | 6.60254 | 0.388793 |
| 15 | 6.60254 | 0.375022 |
| 16.66667 | 6.60254 | 0.359006 |
| 18.33333 | 6.60254 | 0.340429 |
| 20 | 6.60254 | 0.318844 |
| 21.66667 | 6.60254 | 0.293587 |
| 23.33333 | 6.60254 | 0.263605 |
| 25 | 6.60254 | 0.227033 |
| 26.66667 | 6.60254 | 0.179899 |
| 28.33333 | 6.60254 | 0.109295 |
| -28.3333 | 9.489292 | 0.039979 |
| -26.6667 | 9.489292 | 0.148379 |
| -25 | 9.489292 | 0.202971 |
| -23.3333 | 9.489292 | 0.243188 |
| -21.6667 | 9.489292 | 0.275402 |
| -20 | 9.489292 | 0.302183 |
| -18.3333 | 9.489292 | 0.324877 |
| -16.6667 | 9.489292 | 0.344294 |
| -15 | 9.489292 | 0.360963 |
| -13.3333 | 9.489292 | 0.37525 |
| -11.6667 | 9.489292 | 0.387419 |
| -10 | 9.489292 | 0.397665 |
| -8.33333 | 9.489292 | 0.406132 |
| -6.66667 | 9.489292 | 0.412931 |
| -5 | 9.489292 | 0.418142 |
| -3.33333 | 9.489292 | 0.421825 |
| -1.66667 | 9.489292 | 0.42402 |
| 0 | 9.489292 | 0.424749 |
| 1.666667 | 9.489292 | 0.42402 |
| 3.333333 | 9.489292 | 0.421825 |
| 5 | 9.489292 | 0.418142 |
| 6.666667 | 9.489292 | 0.412931 |
| 8.333333 | 9.489292 | 0.406132 |
| 10 | 9.489292 | 0.397665 |
| 11.66667 | 9.489292 | 0.387419 |
| 13.33333 | 9.489292 | 0.37525 |
| 15 | 9.489292 | 0.360963 |
| 16.66667 | 9.489292 | 0.344294 |
| 18.33333 | 9.489292 | 0.324877 |
| 20 | 9.489292 | 0.302183 |
| 21.66667 | 9.489292 | 0.275402 |
| 23.33333 | 9.489292 | 0.243188 |
| 25 | 9.489292 | 0.202971 |
| 26.66667 | 9.489292 | 0.148379 |
| 28.33333 | 9.489292 | 0.039979 |
| -26.6667 | 12.37604 | 0.089202 |
| -25 | 12.37604 | 0.164735 |
| -23.3333 | 12.37604 | 0.212322 |
| -21.6667 | 12.37604 | 0.248569 |
| -20 | 12.37604 | 0.277947 |
| -18.3333 | 12.37604 | 0.302466 |
| -16.6667 | 12.37604 | 0.323232 |
| -15 | 12.37604 | 0.340932 |
| -13.3333 | 12.37604 | 0.356024 |
| -11.6667 | 12.37604 | 0.368828 |
| -10 | 12.37604 | 0.379576 |
| -8.33333 | 12.37604 | 0.388438 |
| -6.66667 | 12.37604 | 0.395541 |
| -5 | 12.37604 | 0.400978 |
| -3.33333 | 12.37604 | 0.404817 |
| -1.66667 | 12.37604 | 0.407104 |
| 0 | 12.37604 | 0.407863 |
| 1.666667 | 12.37604 | 0.407104 |
| 3.333333 | 12.37604 | 0.404817 |
| 5 | 12.37604 | 0.400978 |
| 6.666667 | 12.37604 | 0.395541 |
| 8.333333 | 12.37604 | 0.388438 |
| 10 | 12.37604 | 0.379576 |
| 11.66667 | 12.37604 | 0.368828 |
| 13.33333 | 12.37604 | 0.356024 |
| 15 | 12.37604 | 0.340932 |
| 16.66667 | 12.37604 | 0.323232 |
| 18.33333 | 12.37604 | 0.302466 |
| 20 | 12.37604 | 0.277947 |
| 21.66667 | 12.37604 | 0.248569 |
| 23.33333 | 12.37604 | 0.212322 |
| 25 | 12.37604 | 0.164735 |
| 26.66667 | 12.37604 | 0.089202 |
| -25 | 15.26279 | 0.096777 |
| -23.3333 | 15.26279 | 0.165254 |
| -21.6667 | 15.26279 | 0.209797 |
| -20 | 15.26279 | 0.243891 |
| -18.3333 | 15.26279 | 0.271503 |
| -16.6667 | 15.26279 | 0.294461 |
| -15 | 15.26279 | 0.313788 |
| -13.3333 | 15.26279 | 0.330123 |
| -11.6667 | 15.26279 | 0.343893 |
| -10 | 15.26279 | 0.355395 |
| -8.33333 | 15.26279 | 0.364845 |
| -6.66667 | 15.26279 | 0.372398 |
| -5 | 15.26279 | 0.378169 |
| -3.33333 | 15.26279 | 0.382237 |
| -1.66667 | 15.26279 | 0.384658 |
| 0 | 15.26279 | 0.385461 |
| 1.666667 | 15.26279 | 0.384658 |
| 3.333333 | 15.26279 | 0.382237 |
| 5 | 15.26279 | 0.378169 |
| 6.666667 | 15.26279 | 0.372398 |
| 8.333333 | 15.26279 | 0.364845 |
| 10 | 15.26279 | 0.355395 |
| 11.66667 | 15.26279 | 0.343893 |
| 13.33333 | 15.26279 | 0.330123 |
| 15 | 15.26279 | 0.313788 |
| 16.66667 | 15.26279 | 0.294461 |
| 18.33333 | 15.26279 | 0.271503 |
| 20 | 15.26279 | 0.243891 |
| 21.66667 | 15.26279 | 0.209797 |
| 23.33333 | 15.26279 | 0.165254 |
| 25 | 15.26279 | 0.096777 |
| -23.3333 | 18.14955 | 0.076319 |
| -21.6667 | 18.14955 | 0.150101 |
| -20 | 18.14955 | 0.194932 |
| -18.3333 | 18.14955 | 0.228538 |
| -16.6667 | 18.14955 | 0.255388 |
| -15 | 18.14955 | 0.27745 |
| -13.3333 | 18.14955 | 0.295799 |
| -11.6667 | 18.14955 | 0.311092 |
| -10 | 18.14955 | 0.323762 |
| -8.33333 | 18.14955 | 0.334107 |
| -6.66667 | 18.14955 | 0.342339 |
| -5 | 18.14955 | 0.348608 |
| -3.33333 | 18.14955 | 0.353017 |
| -1.66667 | 18.14955 | 0.355636 |
| 0 | 18.14955 | 0.356505 |
| 1.666667 | 18.14955 | 0.355636 |
| 3.333333 | 18.14955 | 0.353017 |
| 5 | 18.14955 | 0.348608 |
| 6.666667 | 18.14955 | 0.342339 |
| 8.333333 | 18.14955 | 0.334107 |
| 10 | 18.14955 | 0.323762 |
| 11.66667 | 18.14955 | 0.311092 |
| 13.33333 | 18.14955 | 0.295799 |
| 15 | 18.14955 | 0.27745 |
| 16.66667 | 18.14955 | 0.255388 |
| 18.33333 | 18.14955 | 0.228538 |
| 20 | 18.14955 | 0.194932 |
| 21.66667 | 18.14955 | 0.150101 |
| 23.33333 | 18.14955 | 0.076319 |
| -20 | 21.0363 | 0.113146 |
| -18.3333 | 21.0363 | 0.164417 |
| -16.6667 | 21.0363 | 0.200065 |
| -15 | 21.0363 | 0.227557 |
| -13.3333 | 21.0363 | 0.249601 |
| -11.6667 | 21.0363 | 0.267547 |
| -10 | 21.0363 | 0.282179 |
| -8.33333 | 21.0363 | 0.293991 |
| -6.66667 | 21.0363 | 0.303314 |
| -5 | 21.0363 | 0.310372 |
| -3.33333 | 21.0363 | 0.315316 |
| -1.66667 | 21.0363 | 0.318246 |
| 0 | 21.0363 | 0.319217 |
| 1.666667 | 21.0363 | 0.318246 |
| 3.333333 | 21.0363 | 0.315316 |
| 5 | 21.0363 | 0.310372 |
| 6.666667 | 21.0363 | 0.303314 |
| 8.333333 | 21.0363 | 0.293991 |
| 10 | 21.0363 | 0.282179 |
| 11.66667 | 21.0363 | 0.267547 |
| 13.33333 | 21.0363 | 0.249601 |
| 15 | 21.0363 | 0.227557 |
| 16.66667 | 21.0363 | 0.200065 |
| 18.33333 | 21.0363 | 0.164417 |
| 20 | 21.0363 | 0.113146 |
| -16.6667 | 23.92305 | 0.105438 |
| -15 | 23.92305 | 0.151238 |
| -13.3333 | 23.92305 | 0.182733 |
| -11.6667 | 23.92305 | 0.206573 |
| -10 | 23.92305 | 0.225202 |
| -8.33333 | 23.92305 | 0.239837 |
| -6.66667 | 23.92305 | 0.251178 |
| -5 | 23.92305 | 0.259657 |
| -3.33333 | 23.92305 | 0.265547 |
| -1.66667 | 23.92305 | 0.269019 |
| 0 | 23.92305 | 0.270167 |
| 1.666667 | 23.92305 | 0.269019 |
| 3.333333 | 23.92305 | 0.265547 |
| 5 | 23.92305 | 0.259657 |
| 6.666667 | 23.92305 | 0.251178 |
| 8.333333 | 23.92305 | 0.239837 |
| 10 | 23.92305 | 0.225202 |
| 11.66667 | 23.92305 | 0.206573 |
| 13.33333 | 23.92305 | 0.182733 |
| 15 | 23.92305 | 0.151238 |
| 16.66667 | 23.92305 | 0.105438 |
| -13.3333 | 26.8098 | 0.027749 |
| -11.6667 | 26.8098 | 0.100254 |
| -10 | 26.8098 | 0.134516 |
| -8.33333 | 26.8098 | 0.157799 |
| -6.66667 | 26.8098 | 0.174554 |
| -5 | 26.8098 | 0.186548 |
| -3.33333 | 26.8098 | 0.194663 |
| -1.66667 | 26.8098 | 0.199374 |
| 0 | 26.8098 | 0.20092 |
| 1.666667 | 26.8098 | 0.199374 |
| 3.333333 | 26.8098 | 0.194663 |
| 5 | 26.8098 | 0.186548 |
| 6.666667 | 26.8098 | 0.174554 |
| 8.333333 | 26.8098 | 0.157799 |
| 10 | 26.8098 | 0.134516 |
| 11.66667 | 26.8098 | 0.100254 |
| 13.33333 | 26.8098 | 0.027749 |
| -3.33333 | 29.69655 | 0.039497 |
| -1.66667 | 29.69655 | 0.058449 |
| 0 | 29.69655 | 0.063521 |
| 1.666667 | 29.69655 | 0.058449 |
| 3.333333 | 29.69655 | 0.039497 |
| -4.16667 | -29.4819 | 0.054754 |
| -2.5 | -29.4819 | 0.073979 |
| -0.83333 | -29.4819 | 0.081917 |
| 0.833333 | -29.4819 | 0.081917 |
| 2.5 | -29.4819 | 0.073979 |
| 4.166667 | -29.4819 | 0.054754 |
| -12.5 | -26.5951 | 0.090106 |
| -10.8333 | -26.5951 | 0.129543 |
| -9.16667 | -26.5951 | 0.155583 |
| -7.5 | -26.5951 | 0.174337 |
| -5.83333 | -26.5951 | 0.187998 |
| -4.16667 | -26.5951 | 0.197625 |
| -2.5 | -26.5951 | 0.203791 |
| -0.83333 | -26.5951 | 0.206804 |
| 0.833333 | -26.5951 | 0.206804 |
| 2.5 | -26.5951 | 0.203791 |
| 4.166667 | -26.5951 | 0.197625 |
| 5.833333 | -26.5951 | 0.187998 |
| 7.5 | -26.5951 | 0.174337 |
| 9.166667 | -26.5951 | 0.155583 |
| 10.83333 | -26.5951 | 0.129543 |
| 12.5 | -26.5951 | 0.090106 |
| -17.5 | -23.7083 | 0.083982 |
| -15.8333 | -23.7083 | 0.139383 |
| -14.1667 | -23.7083 | 0.174828 |
| -12.5 | -23.7083 | 0.201158 |
| -10.8333 | -23.7083 | 0.221645 |
| -9.16667 | -23.7083 | 0.237806 |
| -7.5 | -23.7083 | 0.250477 |
| -5.83333 | -23.7083 | 0.26017 |
| -4.16667 | -23.7083 | 0.26721 |
| -2.5 | -23.7083 | 0.271801 |
| -0.83333 | -23.7083 | 0.274068 |
| 0.833333 | -23.7083 | 0.274068 |
| 2.5 | -23.7083 | 0.271801 |
| 4.166667 | -23.7083 | 0.26721 |
| 5.833333 | -23.7083 | 0.26017 |
| 7.5 | -23.7083 | 0.250477 |
| 9.166667 | -23.7083 | 0.237806 |
| 10.83333 | -23.7083 | 0.221645 |
| 12.5 | -23.7083 | 0.201158 |
| 14.16667 | -23.7083 | 0.174828 |
| 15.83333 | -23.7083 | 0.139383 |
| 17.5 | -23.7083 | 0.083982 |
| -20.8333 | -20.8216 | 0.084996 |
| -19.1667 | -20.8216 | 0.148573 |
| -17.5 | -20.8216 | 0.188907 |
| -15.8333 | -20.8216 | 0.219227 |
| -14.1667 | -20.8216 | 0.243306 |
| -12.5 | -20.8216 | 0.262864 |
| -10.8333 | -20.8216 | 0.278854 |
| -9.16667 | -20.8216 | 0.291864 |
| -7.5 | -20.8216 | 0.302278 |
| -5.83333 | -20.8216 | 0.310357 |
| -4.16667 | -20.8216 | 0.316281 |
| -2.5 | -20.8216 | 0.32017 |
| -0.83333 | -20.8216 | 0.322097 |
| 0.833333 | -20.8216 | 0.322097 |
| 2.5 | -20.8216 | 0.32017 |
| 4.166667 | -20.8216 | 0.316281 |
| 5.833333 | -20.8216 | 0.310357 |
| 7.5 | -20.8216 | 0.302278 |
| 9.166667 | -20.8216 | 0.291864 |
| 10.83333 | -20.8216 | 0.278854 |
| 12.5 | -20.8216 | 0.262864 |
| 14.16667 | -20.8216 | 0.243306 |
| 15.83333 | -20.8216 | 0.219227 |
| 17.5 | -20.8216 | 0.188907 |
| 19.16667 | -20.8216 | 0.148573 |
| 20.83333 | -20.8216 | 0.084996 |
| -22.5 | -17.9348 | 0.12672 |
| -20.8333 | -17.9348 | 0.17929 |
| -19.1667 | -17.9348 | 0.216782 |
| -17.5 | -17.9348 | 0.246184 |
| -15.8333 | -17.9348 | 0.27015 |
| -14.1667 | -17.9348 | 0.290031 |
| -12.5 | -17.9348 | 0.306623 |
| -10.8333 | -17.9348 | 0.320437 |
| -9.16667 | -17.9348 | 0.331821 |
| -7.5 | -17.9348 | 0.341017 |
| -5.83333 | -17.9348 | 0.348198 |
| -4.16667 | -17.9348 | 0.353489 |
| -2.5 | -17.9348 | 0.356973 |
| -0.83333 | -17.9348 | 0.358702 |
| 0.833333 | -17.9348 | 0.358702 |
| 2.5 | -17.9348 | 0.356973 |
| 4.166667 | -17.9348 | 0.353489 |
| 5.833333 | -17.9348 | 0.348198 |
| 7.5 | -17.9348 | 0.341017 |
| 9.166667 | -17.9348 | 0.331821 |
| 10.83333 | -17.9348 | 0.320437 |
| 12.5 | -17.9348 | 0.306623 |
| 14.16667 | -17.9348 | 0.290031 |
| 15.83333 | -17.9348 | 0.27015 |
| 17.5 | -17.9348 | 0.246184 |
| 19.16667 | -17.9348 | 0.216782 |
| 20.83333 | -17.9348 | 0.17929 |
| 22.5 | -17.9348 | 0.12672 |
| -25.8333 | -15.0481 | 0.037143 |
| -24.1667 | -15.0481 | 0.141215 |
| -22.5 | -15.0481 | 0.193044 |
| -20.8333 | -15.0481 | 0.230983 |
| -19.1667 | -15.0481 | 0.261156 |
| -17.5 | -15.0481 | 0.286033 |
| -15.8333 | -15.0481 | 0.306903 |
| -14.1667 | -15.0481 | 0.32454 |
| -12.5 | -15.0481 | 0.33945 |
| -10.8333 | -15.0481 | 0.351978 |
| -9.16667 | -15.0481 | 0.362372 |
| -7.5 | -15.0481 | 0.370811 |
| -5.83333 | -15.0481 | 0.377426 |
| -4.16667 | -15.0481 | 0.382312 |
| -2.5 | -15.0481 | 0.385535 |
| -0.83333 | -15.0481 | 0.387137 |
| 0.833333 | -15.0481 | 0.387137 |
| 2.5 | -15.0481 | 0.385535 |
| 4.166667 | -15.0481 | 0.382312 |
| 5.833333 | -15.0481 | 0.377426 |
| 7.5 | -15.0481 | 0.370811 |
| 9.166667 | -15.0481 | 0.362372 |
| 10.83333 | -15.0481 | 0.351978 |
| 12.5 | -15.0481 | 0.33945 |
| 14.16667 | -15.0481 | 0.32454 |
| 15.83333 | -15.0481 | 0.306903 |
| 17.5 | -15.0481 | 0.286033 |
| 19.16667 | -15.0481 | 0.261156 |
| 20.83333 | -15.0481 | 0.230983 |
| 22.5 | -15.0481 | 0.193044 |
| 24.16667 | -15.0481 | 0.141215 |
| 25.83333 | -15.0481 | 0.037143 |
| -25.8333 | -12.1613 | 0.137388 |
| -24.1667 | -12.1613 | 0.193487 |
| -22.5 | -12.1613 | 0.234012 |
| -20.8333 | -12.1613 | 0.266174 |
| -19.1667 | -12.1613 | 0.292743 |
| -17.5 | -12.1613 | 0.315136 |
| -15.8333 | -12.1613 | 0.334193 |
| -14.1667 | -12.1613 | 0.35046 |
| -12.5 | -12.1613 | 0.36431 |
| -10.8333 | -12.1613 | 0.376011 |
| -9.16667 | -12.1613 | 0.385758 |
| -7.5 | -12.1613 | 0.393696 |
| -5.83333 | -12.1613 | 0.399933 |
| -4.16667 | -12.1613 | 0.404547 |
| -2.5 | -12.1613 | 0.407595 |
| -0.83333 | -12.1613 | 0.40911 |
| 0.833333 | -12.1613 | 0.40911 |
| 2.5 | -12.1613 | 0.407595 |
| 4.166667 | -12.1613 | 0.404547 |
| 5.833333 | -12.1613 | 0.399933 |
| 7.5 | -12.1613 | 0.393696 |
| 9.166667 | -12.1613 | 0.385758 |
| 10.83333 | -12.1613 | 0.376011 |
| 12.5 | -12.1613 | 0.36431 |
| 14.16667 | -12.1613 | 0.35046 |
| 15.83333 | -12.1613 | 0.334193 |
| 17.5 | -12.1613 | 0.315136 |
| 19.16667 | -12.1613 | 0.292743 |
| 20.83333 | -12.1613 | 0.266174 |
| 22.5 | -12.1613 | 0.234012 |
| 24.16667 | -12.1613 | 0.193487 |
| 25.83333 | -12.1613 | 0.137388 |
| -27.5 | -9.27459 | 0.113399 |
| -25.8333 | -9.27459 | 0.180717 |
| -24.1667 | -9.27459 | 0.22632 |
| -22.5 | -9.27459 | 0.261811 |
| -20.8333 | -9.27459 | 0.290916 |
| -19.1667 | -9.27459 | 0.315407 |
| -17.5 | -9.27459 | 0.336294 |
| -15.8333 | -9.27459 | 0.354215 |
| -14.1667 | -9.27459 | 0.369602 |
| -12.5 | -9.27459 | 0.38276 |
| -10.8333 | -9.27459 | 0.393913 |
| -9.16667 | -9.27459 | 0.403227 |
| -7.5 | -9.27459 | 0.410828 |
| -5.83333 | -9.27459 | 0.416809 |
| -4.16667 | -9.27459 | 0.421238 |
| -2.5 | -9.27459 | 0.424166 |
| -0.83333 | -9.27459 | 0.425622 |
| 0.833333 | -9.27459 | 0.425622 |
| 2.5 | -9.27459 | 0.424166 |
| 4.166667 | -9.27459 | 0.421238 |
| 5.833333 | -9.27459 | 0.416809 |
| 7.5 | -9.27459 | 0.410828 |
| 9.166667 | -9.27459 | 0.403227 |
| 10.83333 | -9.27459 | 0.393913 |
| 12.5 | -9.27459 | 0.38276 |
| 14.16667 | -9.27459 | 0.369602 |
| 15.83333 | -9.27459 | 0.354215 |
| 17.5 | -9.27459 | 0.336294 |
| 19.16667 | -9.27459 | 0.315407 |
| 20.83333 | -9.27459 | 0.290916 |
| 22.5 | -9.27459 | 0.261811 |
| 24.16667 | -9.27459 | 0.22632 |
| 25.83333 | -9.27459 | 0.180717 |
| 27.5 | -9.27459 | 0.113399 |
| -29.1667 | -6.38784 | 0.043515 |
| -27.5 | -6.38784 | 0.151428 |
| -25.8333 | -6.38784 | 0.206712 |
| -24.1667 | -6.38784 | 0.247572 |
| -22.5 | -6.38784 | 0.280386 |
| -20.8333 | -6.38784 | 0.307739 |
| -19.1667 | -6.38784 | 0.330988 |
| -17.5 | -6.38784 | 0.350948 |
| -15.8333 | -6.38784 | 0.368157 |
| -14.1667 | -6.38784 | 0.382984 |
| -12.5 | -6.38784 | 0.395697 |
| -10.8333 | -6.38784 | 0.406495 |
| -9.16667 | -6.38784 | 0.415528 |
| -7.5 | -6.38784 | 0.422907 |
| -5.83333 | -6.38784 | 0.42872 |
| -4.16667 | -6.38784 | 0.433028 |
| -2.5 | -6.38784 | 0.435876 |
| -0.83333 | -6.38784 | 0.437293 |
| 0.833333 | -6.38784 | 0.437293 |
| 2.5 | -6.38784 | 0.435876 |
| 4.166667 | -6.38784 | 0.433028 |
| 5.833333 | -6.38784 | 0.42872 |
| 7.5 | -6.38784 | 0.422907 |
| 9.166667 | -6.38784 | 0.415528 |
| 10.83333 | -6.38784 | 0.406495 |
| 12.5 | -6.38784 | 0.395697 |
| 14.16667 | -6.38784 | 0.382984 |
| 15.83333 | -6.38784 | 0.368157 |
| 17.5 | -6.38784 | 0.350948 |
| 19.16667 | -6.38784 | 0.330988 |
| 20.83333 | -6.38784 | 0.307739 |
| 22.5 | -6.38784 | 0.280386 |
| 24.16667 | -6.38784 | 0.247572 |
| 25.83333 | -6.38784 | 0.206712 |
| 27.5 | -6.38784 | 0.151428 |
| 29.16667 | -6.38784 | 0.043515 |
| -29.1667 | -3.50109 | 0.090841 |
| -27.5 | -3.50109 | 0.17114 |
| -25.8333 | -3.50109 | 0.221559 |
| -24.1667 | -3.50109 | 0.260097 |
| -22.5 | -3.50109 | 0.291504 |
| -20.8333 | -3.50109 | 0.317902 |
| -19.1667 | -3.50109 | 0.340458 |
| -17.5 | -3.50109 | 0.359894 |
| -15.8333 | -3.50109 | 0.376694 |
| -14.1667 | -3.50109 | 0.391197 |
| -12.5 | -3.50109 | 0.403652 |
| -10.8333 | -3.50109 | 0.414243 |
| -9.16667 | -3.50109 | 0.42311 |
| -7.5 | -3.50109 | 0.430359 |
| -5.83333 | -3.50109 | 0.436072 |
| -4.16667 | -3.50109 | 0.440308 |
| -2.5 | -3.50109 | 0.44311 |
| -0.83333 | -3.50109 | 0.444504 |
| 0.833333 | -3.50109 | 0.444504 |
| 2.5 | -3.50109 | 0.44311 |
| 4.166667 | -3.50109 | 0.440308 |
| 5.833333 | -3.50109 | 0.436072 |
| 7.5 | -3.50109 | 0.430359 |
| 9.166667 | -3.50109 | 0.42311 |
| 10.83333 | -3.50109 | 0.414243 |
| 12.5 | -3.50109 | 0.403652 |
| 14.16667 | -3.50109 | 0.391197 |
| 15.83333 | -3.50109 | 0.376694 |
| 17.5 | -3.50109 | 0.359894 |
| 19.16667 | -3.50109 | 0.340458 |
| 20.83333 | -3.50109 | 0.317902 |
| 22.5 | -3.50109 | 0.291504 |
| 24.16667 | -3.50109 | 0.260097 |
| 25.83333 | -3.50109 | 0.221559 |
| 27.5 | -3.50109 | 0.17114 |
| 29.16667 | -3.50109 | 0.090841 |
| -29.1667 | -0.61434 | 0.104395 |
| -27.5 | -0.61434 | 0.178704 |
| -25.8333 | -0.61434 | 0.227453 |
| -24.1667 | -0.61434 | 0.265135 |
| -22.5 | -0.61434 | 0.296009 |
| -20.8333 | -0.61434 | 0.322037 |
| -19.1667 | -0.61434 | 0.344322 |
| -17.5 | -0.61434 | 0.363551 |
| -15.8333 | -0.61434 | 0.38019 |
| -14.1667 | -0.61434 | 0.394565 |
| -12.5 | -0.61434 | 0.406917 |
| -10.8333 | -0.61434 | 0.417425 |
| -9.16667 | -0.61434 | 0.426225 |
| -7.5 | -0.61434 | 0.433423 |
| -5.83333 | -0.61434 | 0.439096 |
| -4.16667 | -0.61434 | 0.443303 |
| -2.5 | -0.61434 | 0.446086 |
| -0.83333 | -0.61434 | 0.447471 |
| 0.833333 | -0.61434 | 0.447471 |
| 2.5 | -0.61434 | 0.446086 |
| 4.166667 | -0.61434 | 0.443303 |
| 5.833333 | -0.61434 | 0.439096 |
| 7.5 | -0.61434 | 0.433423 |
| 9.166667 | -0.61434 | 0.426225 |
| 10.83333 | -0.61434 | 0.417425 |
| 12.5 | -0.61434 | 0.406917 |
| 14.16667 | -0.61434 | 0.394565 |
| 15.83333 | -0.61434 | 0.38019 |
| 17.5 | -0.61434 | 0.363551 |
| 19.16667 | -0.61434 | 0.344322 |
| 20.83333 | -0.61434 | 0.322037 |
| 22.5 | -0.61434 | 0.296009 |
| 24.16667 | -0.61434 | 0.265135 |
| 25.83333 | -0.61434 | 0.227453 |
| 27.5 | -0.61434 | 0.178704 |
| 29.16667 | -0.61434 | 0.104395 |
| -29.1667 | 2.272413 | 0.099158 |
| -27.5 | 2.272413 | 0.175696 |
| -25.8333 | 2.272413 | 0.225097 |
| -24.1667 | 2.272413 | 0.263117 |
| -22.5 | 2.272413 | 0.294202 |
| -20.8333 | 2.272413 | 0.320378 |
| -19.1667 | 2.272413 | 0.34277 |
| -17.5 | 2.272413 | 0.362082 |
| -15.8333 | 2.272413 | 0.378785 |
| -14.1667 | 2.272413 | 0.393212 |
| -12.5 | 2.272413 | 0.405604 |
| -10.8333 | 2.272413 | 0.416146 |
| -9.16667 | 2.272413 | 0.424973 |
| -7.5 | 2.272413 | 0.432191 |
| -5.83333 | 2.272413 | 0.43788 |
| -4.16667 | 2.272413 | 0.442099 |
| -2.5 | 2.272413 | 0.444889 |
| -0.83333 | 2.272413 | 0.446278 |
| 0.833333 | 2.272413 | 0.446278 |
| 2.5 | 2.272413 | 0.444889 |
| 4.166667 | 2.272413 | 0.442099 |
| 5.833333 | 2.272413 | 0.43788 |
| 7.5 | 2.272413 | 0.432191 |
| 9.166667 | 2.272413 | 0.424973 |
| 10.83333 | 2.272413 | 0.416146 |
| 12.5 | 2.272413 | 0.405604 |
| 14.16667 | 2.272413 | 0.393212 |
| 15.83333 | 2.272413 | 0.378785 |
| 17.5 | 2.272413 | 0.362082 |
| 19.16667 | 2.272413 | 0.34277 |
| 20.83333 | 2.272413 | 0.320378 |
| 22.5 | 2.272413 | 0.294202 |
| 24.16667 | 2.272413 | 0.263117 |
| 25.83333 | 2.272413 | 0.225097 |
| 27.5 | 2.272413 | 0.175696 |
| 29.16667 | 2.272413 | 0.099158 |
| -29.1667 | 5.159165 | 0.07109 |
| -27.5 | 5.159165 | 0.161526 |
| -25.8333 | 5.159165 | 0.214219 |
| -24.1667 | 5.159165 | 0.253874 |
| -22.5 | 5.159165 | 0.285966 |
| -20.8333 | 5.159165 | 0.312831 |
| -19.1667 | 5.159165 | 0.335727 |
| -17.5 | 5.159165 | 0.355422 |
| -15.8333 | 5.159165 | 0.372424 |
| -14.1667 | 5.159165 | 0.387088 |
| -12.5 | 5.159165 | 0.39967 |
| -10.8333 | 5.159165 | 0.410364 |
| -9.16667 | 5.159165 | 0.419313 |
| -7.5 | 5.159165 | 0.426627 |
| -5.83333 | 5.159165 | 0.432389 |
| -4.16667 | 5.159165 | 0.436661 |
| -2.5 | 5.159165 | 0.439486 |
| -0.83333 | 5.159165 | 0.440892 |
| 0.833333 | 5.159165 | 0.440892 |
| 2.5 | 5.159165 | 0.439486 |
| 4.166667 | 5.159165 | 0.436661 |
| 5.833333 | 5.159165 | 0.432389 |
| 7.5 | 5.159165 | 0.426627 |
| 9.166667 | 5.159165 | 0.419313 |
| 10.83333 | 5.159165 | 0.410364 |
| 12.5 | 5.159165 | 0.39967 |
| 14.16667 | 5.159165 | 0.387088 |
| 15.83333 | 5.159165 | 0.372424 |
| 17.5 | 5.159165 | 0.355422 |
| 19.16667 | 5.159165 | 0.335727 |
| 20.83333 | 5.159165 | 0.312831 |
| 22.5 | 5.159165 | 0.285966 |
| 24.16667 | 5.159165 | 0.253874 |
| 25.83333 | 5.159165 | 0.214219 |
| 27.5 | 5.159165 | 0.161526 |
| 29.16667 | 5.159165 | 0.07109 |
| -27.5 | 8.045916 | 0.132664 |
| -25.8333 | 8.045916 | 0.193388 |
| -24.1667 | 8.045916 | 0.236561 |
| -22.5 | 8.045916 | 0.270713 |
| -20.8333 | 8.045916 | 0.298952 |
| -19.1667 | 8.045916 | 0.322834 |
| -17.5 | 8.045916 | 0.34327 |
| -15.8333 | 8.045916 | 0.360844 |
| -14.1667 | 8.045916 | 0.37596 |
| -12.5 | 8.045916 | 0.388903 |
| -10.8333 | 8.045916 | 0.399885 |
| -9.16667 | 8.045916 | 0.409063 |
| -7.5 | 8.045916 | 0.416557 |
| -5.83333 | 8.045916 | 0.422457 |
| -4.16667 | 8.045916 | 0.426828 |
| -2.5 | 8.045916 | 0.429717 |
| -0.83333 | 8.045916 | 0.431155 |
| 0.833333 | 8.045916 | 0.431155 |
| 2.5 | 8.045916 | 0.429717 |
| 4.166667 | 8.045916 | 0.426828 |
| 5.833333 | 8.045916 | 0.422457 |
| 7.5 | 8.045916 | 0.416557 |
| 9.166667 | 8.045916 | 0.409063 |
| 10.83333 | 8.045916 | 0.399885 |
| 12.5 | 8.045916 | 0.388903 |
| 14.16667 | 8.045916 | 0.37596 |
| 15.83333 | 8.045916 | 0.360844 |
| 17.5 | 8.045916 | 0.34327 |
| 19.16667 | 8.045916 | 0.322834 |
| 20.83333 | 8.045916 | 0.298952 |
| 22.5 | 8.045916 | 0.270713 |
| 24.16667 | 8.045916 | 0.236561 |
| 25.83333 | 8.045916 | 0.193388 |
| 27.5 | 8.045916 | 0.132664 |
| -27.5 | 10.93267 | 0.07346 |
| -25.8333 | 10.93267 | 0.158732 |
| -24.1667 | 10.93267 | 0.209183 |
| -22.5 | 10.93267 | 0.247148 |
| -20.8333 | 10.93267 | 0.277793 |
| -19.1667 | 10.93267 | 0.303346 |
| -17.5 | 10.93267 | 0.325009 |
| -15.8333 | 10.93267 | 0.343519 |
| -14.1667 | 10.93267 | 0.359364 |
| -12.5 | 10.93267 | 0.372884 |
| -10.8333 | 10.93267 | 0.384323 |
| -9.16667 | 10.93267 | 0.393865 |
| -7.5 | 10.93267 | 0.401642 |
| -5.83333 | 10.93267 | 0.407758 |
| -4.16667 | 10.93267 | 0.412285 |
| -2.5 | 10.93267 | 0.415275 |
| -0.83333 | 10.93267 | 0.416763 |
| 0.833333 | 10.93267 | 0.416763 |
| 2.5 | 10.93267 | 0.415275 |
| 4.166667 | 10.93267 | 0.412285 |
| 5.833333 | 10.93267 | 0.407758 |
| 7.5 | 10.93267 | 0.401642 |
| 9.166667 | 10.93267 | 0.393865 |
| 10.83333 | 10.93267 | 0.384323 |
| 12.5 | 10.93267 | 0.372884 |
| 14.16667 | 10.93267 | 0.359364 |
| 15.83333 | 10.93267 | 0.343519 |
| 17.5 | 10.93267 | 0.325009 |
| 19.16667 | 10.93267 | 0.303346 |
| 20.83333 | 10.93267 | 0.277793 |
| 22.5 | 10.93267 | 0.247148 |
| 24.16667 | 10.93267 | 0.209183 |
| 25.83333 | 10.93267 | 0.158732 |
| 27.5 | 10.93267 | 0.07346 |
| -25.8333 | 13.81942 | 0.096333 |
| -24.1667 | 13.81942 | 0.166859 |
| -22.5 | 13.81942 | 0.212524 |
| -20.8333 | 13.81942 | 0.247494 |
| -19.1667 | 13.81942 | 0.275868 |
| -17.5 | 13.81942 | 0.299525 |
| -15.8333 | 13.81942 | 0.319515 |
| -14.1667 | 13.81942 | 0.336492 |
| -12.5 | 13.81942 | 0.350894 |
| -10.8333 | 13.81942 | 0.363027 |
| -9.16667 | 13.81942 | 0.373113 |
| -7.5 | 13.81942 | 0.381315 |
| -5.83333 | 13.81942 | 0.387751 |
| -4.16667 | 13.81942 | 0.392509 |
| -2.5 | 13.81942 | 0.395649 |
| -0.83333 | 13.81942 | 0.39721 |
| 0.833333 | 13.81942 | 0.39721 |
| 2.5 | 13.81942 | 0.395649 |
| 4.166667 | 13.81942 | 0.392509 |
| 5.833333 | 13.81942 | 0.387751 |
| 7.5 | 13.81942 | 0.381315 |
| 9.166667 | 13.81942 | 0.373113 |
| 10.83333 | 13.81942 | 0.363027 |
| 12.5 | 13.81942 | 0.350894 |
| 14.16667 | 13.81942 | 0.336492 |
| 15.83333 | 13.81942 | 0.319515 |
| 17.5 | 13.81942 | 0.299525 |
| 19.16667 | 13.81942 | 0.275868 |
| 20.83333 | 13.81942 | 0.247494 |
| 22.5 | 13.81942 | 0.212524 |
| 24.16667 | 13.81942 | 0.166859 |
| 25.83333 | 13.81942 | 0.096333 |
| -24.1667 | 16.70617 | 0.090631 |
| -22.5 | 16.70617 | 0.159807 |
| -20.8333 | 16.70617 | 0.204023 |
| -19.1667 | 16.70617 | 0.237645 |
| -17.5 | 16.70617 | 0.264739 |
| -15.8333 | 16.70617 | 0.287161 |
| -14.1667 | 16.70617 | 0.305939 |
| -12.5 | 16.70617 | 0.321712 |
| -10.8333 | 16.70617 | 0.334904 |
| -9.16667 | 16.70617 | 0.345811 |
| -7.5 | 16.70617 | 0.354645 |
| -5.83333 | 16.70617 | 0.361556 |
| -4.16667 | 16.70617 | 0.366654 |
| -2.5 | 16.70617 | 0.370013 |
| -0.83333 | 16.70617 | 0.371682 |
| 0.833333 | 16.70617 | 0.371682 |
| 2.5 | 16.70617 | 0.370013 |
| 4.166667 | 16.70617 | 0.366654 |
| 5.833333 | 16.70617 | 0.361556 |
| 7.5 | 16.70617 | 0.354645 |
| 9.166667 | 16.70617 | 0.345811 |
| 10.83333 | 16.70617 | 0.334904 |
| 12.5 | 16.70617 | 0.321712 |
| 14.16667 | 16.70617 | 0.305939 |
| 15.83333 | 16.70617 | 0.287161 |
| 17.5 | 16.70617 | 0.264739 |
| 19.16667 | 16.70617 | 0.237645 |
| 20.83333 | 16.70617 | 0.204023 |
| 22.5 | 16.70617 | 0.159807 |
| 24.16667 | 16.70617 | 0.090631 |
| -22.5 | 19.59292 | 0.046882 |
| -20.8333 | 19.59292 | 0.135222 |
| -19.1667 | 19.59292 | 0.182029 |
| -17.5 | 19.59292 | 0.216209 |
| -15.8333 | 19.59292 | 0.243148 |
| -14.1667 | 19.59292 | 0.265063 |
| -12.5 | 19.59292 | 0.283122 |
| -10.8333 | 19.59292 | 0.298027 |
| -9.16667 | 19.59292 | 0.310234 |
| -7.5 | 19.59292 | 0.320051 |
| -5.83333 | 19.59292 | 0.327692 |
| -4.16667 | 19.59292 | 0.333309 |
| -2.5 | 19.59292 | 0.337001 |
| -0.83333 | 19.59292 | 0.338832 |
| 0.833333 | 19.59292 | 0.338832 |
| 2.5 | 19.59292 | 0.337001 |
| 4.166667 | 19.59292 | 0.333309 |
| 5.833333 | 19.59292 | 0.327692 |
| 7.5 | 19.59292 | 0.320051 |
| 9.166667 | 19.59292 | 0.310234 |
| 10.83333 | 19.59292 | 0.298027 |
| 12.5 | 19.59292 | 0.283122 |
| 14.16667 | 19.59292 | 0.265063 |
| 15.83333 | 19.59292 | 0.243148 |
| 17.5 | 19.59292 | 0.216209 |
| 19.16667 | 19.59292 | 0.182029 |
| 20.83333 | 19.59292 | 0.135222 |
| 22.5 | 19.59292 | 0.046882 |
| -19.1667 | 22.47967 | 0.077985 |
| -17.5 | 22.47967 | 0.140334 |
| -15.8333 | 22.47967 | 0.179076 |
| -14.1667 | 22.47967 | 0.207859 |
| -12.5 | 22.47967 | 0.230446 |
| -10.8333 | 22.47967 | 0.24853 |
| -9.16667 | 22.47967 | 0.263044 |
| -7.5 | 22.47967 | 0.274553 |
| -5.83333 | 22.47967 | 0.283424 |
| -4.16667 | 22.47967 | 0.289899 |
| -2.5 | 22.47967 | 0.294137 |
| -0.83333 | 22.47967 | 0.296233 |
| 0.833333 | 22.47967 | 0.296233 |
| 2.5 | 22.47967 | 0.294137 |
| 4.166667 | 22.47967 | 0.289899 |
| 5.833333 | 22.47967 | 0.283424 |
| 7.5 | 22.47967 | 0.274553 |
| 9.166667 | 22.47967 | 0.263044 |
| 10.83333 | 22.47967 | 0.24853 |
| 12.5 | 22.47967 | 0.230446 |
| 14.16667 | 22.47967 | 0.207859 |
| 15.83333 | 22.47967 | 0.179076 |
| 17.5 | 22.47967 | 0.140334 |
| 19.16667 | 22.47967 | 0.077985 |
| -15.8333 | 25.36642 | 0.036098 |
| -14.1667 | 25.36642 | 0.111536 |
| -12.5 | 25.36642 | 0.149465 |
| -10.8333 | 25.36642 | 0.176074 |
| -9.16667 | 25.36642 | 0.196028 |
| -7.5 | 25.36642 | 0.211221 |
| -5.83333 | 25.36642 | 0.22263 |
| -4.16667 | 25.36642 | 0.230817 |
| -2.5 | 25.36642 | 0.236117 |
| -0.83333 | 25.36642 | 0.238723 |
| 0.833333 | 25.36642 | 0.238723 |
| 2.5 | 25.36642 | 0.236117 |
| 4.166667 | 25.36642 | 0.230817 |
| 5.833333 | 25.36642 | 0.22263 |
| 7.5 | 25.36642 | 0.211221 |
| 9.166667 | 25.36642 | 0.196028 |
| 10.83333 | 25.36642 | 0.176074 |
| 12.5 | 25.36642 | 0.149465 |
| 14.16667 | 25.36642 | 0.111536 |
| 15.83333 | 25.36642 | 0.036098 |
| -9.16667 | 28.25318 | 0.062843 |
| -7.5 | 28.25318 | 0.100681 |
| -5.83333 | 28.25318 | 0.122827 |
| -4.16667 | 28.25318 | 0.137109 |
| -2.5 | 28.25318 | 0.145855 |
| -0.83333 | 28.25318 | 0.150037 |
| 0.833333 | 28.25318 | 0.150037 |
| 2.5 | 28.25318 | 0.145855 |
| 4.166667 | 28.25318 | 0.137109 |
| 5.833333 | 28.25318 | 0.122827 |
| 7.5 | 28.25318 | 0.100681 |
| 9.166667 | 28.25318 | 0.062843 |
