# Supplementary material for: Effective media properties of hyperuniform disordered composite materials
Source: PLoS One. 2017 Oct 5;12(10):e0185921. doi: 10.1371/journal.pone.0185921 (PMC5628886; doi:10.1371/journal.pone.0185921)
Supplement: S2 File — Positions (x,y-coordinates) and radius of each glass fiber in the hyperuniform Luneburg lens design. (DOCX) [file pone.0185921.s002.docx]

S2File

Glass fiber configuration of hyperuniform Luneburg lens design.

| X-coordinate(cm) | Y-coordinate(cm) | Radius(cm) |
| --- | --- | --- |
| -26.4371 | -12.5641 | 0.110145 |
| -26.1028 | -11.089 | 0.163879 |
| -27.5651 | -10.26 | 0.098977 |
| -24.9809 | -10.1795 | 0.219938 |
| -23.7075 | -10.1771 | 0.256495 |
| -24.7528 | -12.0316 | 0.200028 |
| -23.1805 | -11.6127 | 0.252874 |
| -23.1031 | -12.9359 | 0.236291 |
| -24.9748 | -13.4687 | 0.163173 |
| -25.01 | -14.6408 | 0.129933 |
| -19.6773 | -22.3434 | 0.061739 |
| -20.2415 | -21.059 | 0.114618 |
| -23.2048 | -18.6449 | 0.062474 |
| -24.0692 | -17.0209 | 0.093225 |
| -24.0341 | -15.7732 | 0.143702 |
| -22.4242 | -15.4677 | 0.210532 |
| -20.7774 | -14.8596 | 0.263571 |
| -23.0257 | -14.259 | 0.216184 |
| -21.4165 | -13.5754 | 0.268609 |
| -20.8944 | -12.4024 | 0.294797 |
| -21.6455 | -11.3074 | 0.291931 |
| -19.9234 | -10.8883 | 0.328525 |
| -18.1267 | -10.3556 | 0.360965 |
| -18.6107 | -11.9066 | 0.340018 |
| -19.3204 | -12.9287 | 0.317696 |
| -19.3926 | -14.3277 | 0.299068 |
| -19.9136 | -16.1434 | 0.261082 |
| -21.4109 | -16.5621 | 0.216683 |
| -22.4205 | -17.4336 | 0.161907 |
| -21.294 | -19.0194 | 0.154309 |
| -20.4718 | -18.0727 | 0.208104 |
| -19.1246 | -17.4652 | 0.253654 |
| -19.9442 | -19.8108 | 0.175528 |
| -18.5603 | -18.8252 | 0.237605 |
| -17.3261 | -17.8777 | 0.280446 |
| -18.2581 | -20.1857 | 0.21138 |
| -18.3301 | -21.7359 | 0.160313 |
| -17.4298 | -22.4903 | 0.159284 |
| -17.577 | -23.8895 | 0.075564 |
| -16.2275 | -24.5297 | 0.099068 |
| -11.7295 | -26.4493 | 0.132825 |
| -13.9403 | -25.8864 | 0.099912 |
| -12.593 | -25.3167 | 0.16794 |
| -10.3437 | -26.4467 | 0.162072 |
| -10.1975 | -24.5561 | 0.232761 |
| -11.3588 | -24.407 | 0.221808 |
| -13.6066 | -24.071 | 0.194985 |
| -14.9177 | -23.9223 | 0.171829 |
| -15.7814 | -22.7519 | 0.193435 |
| -16.0836 | -21.3536 | 0.22809 |
| -16.91 | -20.0697 | 0.243516 |
| -15.5998 | -19.6892 | 0.274777 |
| -16.2394 | -18.1781 | 0.292999 |
| -16.992 | -16.2891 | 0.311614 |
| -18.3408 | -15.9892 | 0.294011 |
| -17.9317 | -14.4762 | 0.321788 |
| -17.2971 | -13.3408 | 0.344511 |
| -16.9629 | -11.8278 | 0.36413 |
| -15.8777 | -11.3343 | 0.381842 |
| -14.7567 | -9.93331 | 0.40473 |
| -13.2567 | -10.9135 | 0.412147 |
| -14.6033 | -11.8612 | 0.391505 |
| -13.1415 | -12.4633 | 0.400686 |
| -11.6079 | -11.364 | 0.422558 |
| -10.3731 | -10.7568 | 0.435826 |
| -10.1822 | -12.6846 | 0.422328 |
| -11.605 | -12.9519 | 0.409557 |
| -12.764 | -14.0505 | 0.389207 |
| -14.5252 | -13.5246 | 0.376911 |
| -15.8361 | -13.527 | 0.361761 |
| -16.1703 | -15.0399 | 0.340201 |
| -14.2603 | -14.9985 | 0.36387 |
| -15.2693 | -16.2103 | 0.336783 |
| -14.8549 | -17.4949 | 0.323686 |
| -13.3948 | -17.1519 | 0.345957 |
| -14.2529 | -18.9683 | 0.30759 |
| -13.8383 | -20.3285 | 0.287882 |
| -14.5101 | -21.6153 | 0.249763 |
| -13.4974 | -22.3695 | 0.247044 |
| -12.2225 | -23.161 | 0.245191 |
| -10.5382 | -22.5907 | 0.279649 |
| -11.8135 | -21.6102 | 0.286999 |
| -12.3774 | -20.477 | 0.303193 |
| -10.8051 | -20.096 | 0.326339 |
| -11.5196 | -18.5094 | 0.34527 |
| -12.7179 | -18.6629 | 0.330845 |
| -11.8973 | -16.8466 | 0.365009 |
| -13.0231 | -15.6767 | 0.368832 |
| -11.4517 | -14.7664 | 0.393206 |
| -10.7003 | -16.0504 | 0.384937 |
| -10.1358 | -17.486 | 0.371435 |
| -10.1044 | -14.2723 | 0.4084 |
| -28.0187 | -8.03021 | 0.119024 |
| -26.7429 | -9.27544 | 0.166517 |
| -25.3958 | -8.66797 | 0.22473 |
| -24.1985 | -7.98518 | 0.265258 |
| -26.4841 | -7.49801 | 0.199909 |
| -27.8343 | -6.47977 | 0.152879 |
| -28.8849 | -5.46096 | 0.100301 |
| -27.125 | -5.23079 | 0.196008 |
| -26.1526 | -4.51062 | 0.234374 |
| -24.6167 | -4.65894 | 0.276472 |
| -25.6621 | -6.43785 | 0.236944 |
| -24.4264 | -6.28429 | 0.272147 |
| -23.1486 | -8.62592 | 0.285179 |
| -21.9852 | -9.90916 | 0.298983 |
| -21.4256 | -8.73608 | 0.31992 |
| -21.4662 | -7.07264 | 0.330518 |
| -22.7398 | -6.92382 | 0.30663 |
| -22.9672 | -5.52539 | 0.309826 |
| -22.4453 | -4.20115 | 0.325975 |
| -23.9446 | -3.56126 | 0.296868 |
| -25.8558 | -2.99777 | 0.249905 |
| -27.5781 | -3.30348 | 0.189947 |
| -28.6631 | -3.91045 | 0.133124 |
| -29.4898 | -2.43753 | 0.082774 |
| -29.7177 | -0.77444 | 0.067561 |
| -29.7213 | 1.153717 | 0.065567 |
| -28.6385 | 2.932701 | 0.141409 |
| -27.3665 | 3.69125 | 0.196423 |
| -29.0157 | 4.368661 | 0.104636 |
| -28.4563 | 5.655163 | 0.127879 |
| -27.2215 | 6.300219 | 0.182991 |
| -26.4371 | 7.435917 | 0.20229 |
| -28.909 | 7.393438 | 0.051932 |
| -27.8993 | 8.227104 | 0.123052 |
| -26.1028 | 8.91103 | 0.197694 |
| -27.5651 | 9.740023 | 0.112763 |
| -24.9809 | 9.820523 | 0.224472 |
| -23.7075 | 9.82293 | 0.260342 |
| -24.7528 | 7.968402 | 0.250642 |
| -23.1805 | 8.387253 | 0.286449 |
| -23.1031 | 7.064148 | 0.297988 |
| -24.9748 | 6.531309 | 0.256069 |
| -25.01 | 5.35922 | 0.262657 |
| -26.6199 | 5.015912 | 0.216005 |
| -25.7934 | 3.656416 | 0.249254 |
| -26.0155 | 2.294937 | 0.247327 |
| -27.4752 | 1.762878 | 0.19965 |
| -28.3722 | 0.740388 | 0.162843 |
| -28.2195 | -0.77161 | 0.170086 |
| -27.6182 | -1.90469 | 0.193659 |
| -25.4833 | -1.93846 | 0.263228 |
| -26.3844 | -0.73033 | 0.238902 |
| -26.4991 | 0.517086 | 0.23547 |
| -24.8156 | 1.541062 | 0.281253 |
| -24.738 | 0.104536 | 0.284334 |
| -23.9871 | -0.91484 | 0.301472 |
| -23.6849 | -2.23752 | 0.306198 |
| -22.1118 | -2.31016 | 0.337472 |
| -21.2111 | -3.29144 | 0.351138 |
| -21.2075 | -5.1818 | 0.344738 |
| -20.1954 | -5.67138 | 0.359326 |
| -19.0326 | -6.57655 | 0.372568 |
| -20.1541 | -7.71289 | 0.349161 |
| -20.0004 | -9.79199 | 0.336793 |
| -19.0287 | -8.69375 | 0.360242 |
| -17.8688 | -8.04883 | 0.380549 |
| -17.1916 | -9.71107 | 0.378413 |
| -15.8437 | -9.48168 | 0.396145 |
| -15.3598 | -7.89286 | 0.410996 |
| -16.6337 | -7.63062 | 0.398272 |
| -17.8726 | -6.04506 | 0.390777 |
| -18.6994 | -4.49653 | 0.385747 |
| -19.7495 | -3.74237 | 0.373111 |
| -19.6773 | -2.34336 | 0.377361 |
| -20.2415 | -1.05899 | 0.370548 |
| -21.7397 | -0.98621 | 0.345966 |
| -22.6406 | 0.070694 | 0.32976 |
| -23.2048 | 1.355071 | 0.317753 |
| -24.0692 | 2.979146 | 0.295838 |
| -24.0341 | 4.226849 | 0.292353 |
| -22.4242 | 4.53235 | 0.325137 |
| -20.7774 | 5.140378 | 0.352183 |
| -23.0257 | 5.741043 | 0.307497 |
| -21.4165 | 6.424614 | 0.335104 |
| -20.8944 | 7.597624 | 0.337459 |
| -21.6455 | 8.692612 | 0.316074 |
| -19.9234 | 9.111747 | 0.343368 |
| -18.1267 | 9.644444 | 0.366453 |
| -18.6107 | 8.093434 | 0.370161 |
| -19.3204 | 7.071299 | 0.365806 |
| -19.3926 | 5.672296 | 0.371527 |
| -19.9136 | 3.856567 | 0.370326 |
| -21.4109 | 3.437857 | 0.347319 |
| -22.4205 | 2.566384 | 0.331176 |
| -21.294 | 0.980611 | 0.353666 |
| -20.4718 | 1.927345 | 0.365986 |
| -19.1246 | 2.534807 | 0.384914 |
| -19.9442 | 0.189212 | 0.375452 |
| -18.5603 | 1.174815 | 0.394391 |
| -17.3261 | 2.122328 | 0.408778 |
| -18.2581 | -0.18567 | 0.398804 |
| -18.3301 | -1.7359 | 0.396824 |
| -17.4298 | -2.49035 | 0.406953 |
| -17.577 | -3.88949 | 0.402069 |
| -16.2275 | -4.52966 | 0.415874 |
| -16.5245 | -5.92909 | 0.407569 |
| -15.026 | -6.11529 | 0.422793 |
| -13.9741 | -7.81463 | 0.425055 |
| -13.447 | -9.28811 | 0.421492 |
| -12.0227 | -9.85252 | 0.42989 |
| -11.4637 | -8.37699 | 0.442765 |
| -12.7388 | -7.47203 | 0.437498 |
| -11.7295 | -6.44932 | 0.449815 |
| -13.9403 | -5.8864 | 0.433996 |
| -12.593 | -5.31674 | 0.447413 |
| -10.3437 | -6.4467 | 0.45927 |
| -10.414 | -8.94211 | 0.446923 |
| -10.1975 | -4.55607 | 0.466488 |
| -11.3588 | -4.40704 | 0.459303 |
| -13.6066 | -4.07102 | 0.442726 |
| -14.9177 | -3.92227 | 0.431095 |
| -15.7814 | -2.75188 | 0.424963 |
| -16.0836 | -1.35358 | 0.423676 |
| -16.91 | -0.0697 | 0.415164 |
| -15.5998 | 0.310846 | 0.429291 |
| -16.2394 | 1.821925 | 0.42151 |
| -16.992 | 3.710861 | 0.409533 |
| -18.3408 | 4.010769 | 0.392032 |
| -17.9317 | 5.523829 | 0.39218 |
| -17.2971 | 6.659244 | 0.395218 |
| -16.9629 | 8.172164 | 0.391297 |
| -15.8777 | 8.665709 | 0.40098 |
| -14.7567 | 10.06669 | 0.403803 |
| -13.2567 | 9.086542 | 0.424407 |
| -14.6033 | 8.138817 | 0.417341 |
| -13.1415 | 7.536665 | 0.433829 |
| -11.6079 | 8.635973 | 0.440307 |
| -10.3731 | 9.243222 | 0.445468 |
| -10.1822 | 7.315416 | 0.456622 |
| -11.605 | 7.048077 | 0.448197 |
| -12.764 | 5.949477 | 0.443802 |
| -14.5252 | 6.475448 | 0.426186 |
| -15.8361 | 6.47297 | 0.412883 |
| -16.1703 | 4.960051 | 0.41512 |
| -14.2603 | 5.001469 | 0.434193 |
| -15.2693 | 3.789732 | 0.427961 |
| -14.8549 | 2.505071 | 0.434655 |
| -13.3948 | 2.848096 | 0.447199 |
| -14.2529 | 1.031729 | 0.441935 |
| -13.8383 | -0.32855 | 0.445919 |
| -14.5101 | -1.61526 | 0.439085 |
| -13.4974 | -2.36949 | 0.447117 |
| -12.2225 | -3.16103 | 0.455948 |
| -10.5382 | -2.59074 | 0.468583 |
| -11.8135 | -1.61016 | 0.461222 |
| -12.3774 | -0.47701 | 0.457778 |
| -10.8051 | -0.09597 | 0.468885 |
| -11.5196 | 1.490582 | 0.463416 |
| -12.7179 | 1.337088 | 0.454669 |
| -11.8973 | 3.153384 | 0.458371 |
| -13.0231 | 4.323279 | 0.446961 |
| -11.4517 | 5.233621 | 0.45621 |
| -10.7003 | 3.949598 | 0.464876 |
| -10.1358 | 2.513992 | 0.471185 |
| -10.1044 | 5.727662 | 0.463422 |
| -26.7429 | 10.72456 | 0.139973 |
| -25.3958 | 11.33203 | 0.188541 |
| -24.1985 | 12.01482 | 0.218494 |
| -26.4841 | 12.50199 | 0.108957 |
| -24.6167 | 15.34106 | 0.128327 |
| -25.6621 | 13.56215 | 0.127071 |
| -24.4264 | 13.71571 | 0.179845 |
| -23.1486 | 11.37408 | 0.256709 |
| -21.9852 | 10.09084 | 0.297272 |
| -21.4256 | 11.26392 | 0.296914 |
| -21.4662 | 12.92736 | 0.276359 |
| -22.7398 | 13.07618 | 0.243892 |
| -22.9672 | 14.47461 | 0.213897 |
| -22.4453 | 15.79885 | 0.202858 |
| -23.9446 | 16.43874 | 0.125847 |
| -23.6849 | 17.76248 | 0.081248 |
| -22.1118 | 17.68984 | 0.165974 |
| -21.2111 | 16.70856 | 0.219032 |
| -21.2075 | 14.8182 | 0.254452 |
| -20.1954 | 14.32862 | 0.28375 |
| -19.0326 | 13.42345 | 0.31681 |
| -20.1541 | 12.28711 | 0.310216 |
| -20.0004 | 10.20801 | 0.333308 |
| -19.0287 | 11.30626 | 0.339275 |
| -17.8688 | 11.95117 | 0.350585 |
| -17.1916 | 10.28893 | 0.374102 |
| -15.8437 | 10.51833 | 0.38873 |
| -15.3598 | 12.10714 | 0.381129 |
| -16.6337 | 12.36938 | 0.363342 |
| -17.8726 | 13.95494 | 0.329092 |
| -18.6994 | 15.50347 | 0.294972 |
| -19.7495 | 16.25763 | 0.262586 |
| -19.6773 | 17.65664 | 0.237558 |
| -20.2415 | 18.94101 | 0.192139 |
| -21.7397 | 19.0138 | 0.135966 |
| -21.294 | 20.98061 | 0.042321 |
| -20.4718 | 21.92735 | 0.005245 |
| -19.1246 | 22.53481 | 0.086136 |
| -19.9442 | 20.18921 | 0.162976 |
| -18.5603 | 21.17482 | 0.173422 |
| -17.3261 | 22.12233 | 0.176044 |
| -18.2581 | 19.81433 | 0.221023 |
| -18.3301 | 18.2641 | 0.254325 |
| -17.4298 | 17.50966 | 0.285121 |
| -17.577 | 16.11051 | 0.305043 |
| -16.2275 | 15.47034 | 0.333963 |
| -16.5245 | 14.07091 | 0.346994 |
| -15.026 | 13.88471 | 0.36761 |
| -13.9741 | 12.18538 | 0.395139 |
| -13.447 | 10.71189 | 0.411901 |
| -12.0227 | 10.14748 | 0.42796 |
| -11.4637 | 11.62301 | 0.421685 |
| -12.7388 | 12.52798 | 0.403758 |
| -11.7295 | 13.55068 | 0.403071 |
| -13.9403 | 14.11361 | 0.377049 |
| -12.593 | 14.68326 | 0.384181 |
| -10.3437 | 13.5533 | 0.413561 |
| -10.414 | 11.05789 | 0.433431 |
| -10.1975 | 15.44393 | 0.395585 |
| -11.3588 | 15.59297 | 0.384918 |
| -13.6066 | 15.92898 | 0.359774 |
| -14.9177 | 16.07773 | 0.342932 |
| -15.7814 | 17.24812 | 0.31498 |
| -16.0836 | 18.64642 | 0.28709 |
| -16.91 | 19.9303 | 0.246708 |
| -15.5998 | 20.31085 | 0.261767 |
| -16.2394 | 21.82193 | 0.211981 |
| -16.992 | 23.71086 | 0.117359 |
| -10.1822 | 27.31542 | 0.118694 |
| -11.605 | 27.04808 | 0.097298 |
| -12.764 | 25.94948 | 0.133724 |
| -16.1703 | 24.96005 | 0.065995 |
| -14.2603 | 25.00147 | 0.141738 |
| -15.2693 | 23.78973 | 0.168291 |
| -14.8549 | 22.50507 | 0.220272 |
| -13.3948 | 22.8481 | 0.236073 |
| -14.2529 | 21.03173 | 0.267289 |
| -13.8383 | 19.67145 | 0.300422 |
| -14.5101 | 18.38474 | 0.314091 |
| -13.4974 | 17.63051 | 0.337993 |
| -12.2225 | 16.83897 | 0.36208 |
| -10.5382 | 17.40926 | 0.369297 |
| -11.8135 | 18.38984 | 0.344276 |
| -12.3774 | 19.52299 | 0.320371 |
| -10.8051 | 19.90403 | 0.329624 |
| -11.5196 | 21.49058 | 0.292813 |
| -12.7179 | 21.33709 | 0.281838 |
| -11.8973 | 23.15338 | 0.24984 |
| -13.0231 | 24.32328 | 0.197369 |
| -11.4517 | 25.23362 | 0.192578 |
| -10.7003 | 23.9496 | 0.243896 |
| -10.1358 | 22.51399 | 0.2855 |
| -10.1044 | 25.72766 | 0.195376 |
| -9.59297 | -27.3527 | 0.129567 |
| -8.01867 | -28.0302 | 0.118478 |
| -5.39575 | -28.668 | 0.117318 |
| -4.19853 | -27.9852 | 0.16687 |
| -6.48409 | -27.498 | 0.169055 |
| -7.83431 | -26.4798 | 0.196419 |
| -8.8849 | -25.461 | 0.220236 |
| -7.12504 | -25.2308 | 0.244311 |
| -6.1526 | -24.5106 | 0.270864 |
| -4.61674 | -24.6589 | 0.275613 |
| -5.6621 | -26.4379 | 0.217789 |
| -4.42643 | -26.2843 | 0.23066 |
| -3.14864 | -28.6259 | 0.140816 |
| -1.98519 | -29.9092 | 0.020526 |
| -1.42559 | -28.7361 | 0.142365 |
| -1.46616 | -27.0726 | 0.215158 |
| -2.73984 | -26.9238 | 0.216899 |
| -2.96718 | -25.5254 | 0.259363 |
| -2.44532 | -24.2011 | 0.294187 |
| -3.94464 | -23.5613 | 0.304032 |
| -5.85581 | -22.9978 | 0.307477 |
| -7.57808 | -23.3035 | 0.289956 |
| -8.66309 | -23.9104 | 0.266616 |
| -9.48982 | -22.4375 | 0.293315 |
| -9.71765 | -20.7744 | 0.324003 |
| -9.72126 | -18.8463 | 0.35553 |
| -8.63845 | -17.0673 | 0.387189 |
| -7.36646 | -16.3088 | 0.403406 |
| -9.01567 | -15.6313 | 0.40153 |
| -8.45628 | -14.3448 | 0.418084 |
| -7.22154 | -13.6998 | 0.430472 |
| -6.43715 | -12.5641 | 0.443493 |
| -8.90898 | -12.6066 | 0.430974 |
| -7.8993 | -11.7729 | 0.442952 |
| -6.10283 | -11.089 | 0.455693 |
| -9.17497 | -10.5655 | 0.444594 |
| -7.56506 | -10.26 | 0.454988 |
| -4.98094 | -10.1795 | 0.465378 |
| -3.70753 | -10.1771 | 0.468717 |
| -4.75275 | -12.0316 | 0.45349 |
| -3.1805 | -11.6127 | 0.460363 |
| -3.10312 | -12.9359 | 0.450503 |
| -4.97478 | -13.4687 | 0.441316 |
| -5.01004 | -14.6408 | 0.430596 |
| -6.61988 | -14.9841 | 0.421074 |
| -5.79336 | -16.3436 | 0.410156 |
| -6.01553 | -17.7051 | 0.393039 |
| -7.47521 | -18.2371 | 0.378925 |
| -8.37217 | -19.2596 | 0.358932 |
| -8.21953 | -20.7716 | 0.33549 |
| -7.61816 | -21.9047 | 0.318832 |
| -5.48326 | -21.9385 | 0.330287 |
| -6.3844 | -20.7303 | 0.347213 |
| -6.4991 | -19.4829 | 0.366364 |
| -4.81562 | -18.4589 | 0.387911 |
| -4.73802 | -19.8955 | 0.367719 |
| -3.98705 | -20.9148 | 0.35409 |
| -3.68495 | -22.2375 | 0.331676 |
| -2.11178 | -22.3102 | 0.334156 |
| -1.21107 | -23.2914 | 0.316132 |
| -1.20753 | -25.1818 | 0.272436 |
| -0.19537 | -25.6714 | 0.260061 |
| 0.967372 | -26.5766 | 0.232604 |
| -0.1541 | -27.7129 | 0.192469 |
| -0.00039 | -29.792 | 0.059086 |
| 0.971337 | -28.6937 | 0.145794 |
| 2.131178 | -28.0488 | 0.174693 |
| 2.808448 | -29.7111 | 0.051272 |
| 4.156333 | -29.4817 | 0.061685 |
| 4.640249 | -27.8929 | 0.167922 |
| 3.36635 | -27.6306 | 0.187478 |
| 2.127425 | -26.0451 | 0.246875 |
| 1.300555 | -24.4965 | 0.28933 |
| 0.250454 | -23.7424 | 0.307214 |
| 0.322741 | -22.3434 | 0.33536 |
| -0.24146 | -21.059 | 0.357948 |
| -1.73973 | -20.9862 | 0.357984 |
| -2.64058 | -19.9293 | 0.373073 |
| -3.20479 | -18.6449 | 0.390083 |
| -4.06925 | -17.0209 | 0.408238 |
| -4.03413 | -15.7732 | 0.422165 |
| -2.42422 | -15.4677 | 0.428744 |
| -0.77742 | -14.8596 | 0.436437 |
| -3.02573 | -14.259 | 0.439302 |
| -1.41653 | -13.5754 | 0.447587 |
| -0.89438 | -12.4024 | 0.457412 |
| -1.64549 | -11.3074 | 0.464734 |
| 0.07657 | -10.8883 | 0.468346 |
| 1.873325 | -10.3556 | 0.470681 |
| 1.389338 | -11.9066 | 0.460751 |
| 0.679642 | -12.9287 | 0.453408 |
| 0.607355 | -14.3277 | 0.441476 |
| 0.086411 | -16.1434 | 0.423642 |
| -1.41093 | -16.5621 | 0.418416 |
| -2.42054 | -17.4336 | 0.407026 |
| -1.29397 | -19.0194 | 0.388096 |
| -0.47177 | -18.0727 | 0.401102 |
| 0.875403 | -17.4652 | 0.408399 |
| 0.055826 | -19.8108 | 0.377441 |
| 1.439748 | -18.8252 | 0.390602 |
| 2.673928 | -17.8777 | 0.401131 |
| 1.741921 | -20.1857 | 0.370678 |
| 1.669918 | -21.7359 | 0.345296 |
| 2.570207 | -22.4903 | 0.329836 |
| 2.423014 | -23.8895 | 0.301303 |
| 3.772528 | -24.5297 | 0.282375 |
| 3.475523 | -25.9291 | 0.246006 |
| 4.973999 | -26.1153 | 0.232905 |
| 6.02587 | -27.8146 | 0.158974 |
| 8.536287 | -28.377 | 0.078366 |
| 7.261184 | -27.472 | 0.16119 |
| 8.270504 | -26.4493 | 0.192512 |
| 6.059712 | -25.8864 | 0.232859 |
| 7.40696 | -25.3167 | 0.239412 |
| 9.656267 | -26.4467 | 0.17357 |
| 9.80254 | -24.5561 | 0.237476 |
| 8.641212 | -24.407 | 0.25388 |
| 6.393392 | -24.071 | 0.280203 |
| 5.082252 | -23.9223 | 0.291103 |
| 4.218637 | -22.7519 | 0.31989 |
| 3.916393 | -21.3536 | 0.346887 |
| 3.090018 | -20.0697 | 0.369979 |
| 4.400167 | -19.6892 | 0.37199 |
| 3.760633 | -18.1781 | 0.394846 |
| 3.008032 | -16.2891 | 0.419056 |
| 1.659156 | -15.9892 | 0.424373 |
| 2.068308 | -14.4762 | 0.438866 |
| 2.702885 | -13.3408 | 0.447905 |
| 3.037131 | -11.8278 | 0.459096 |
| 4.122349 | -11.3343 | 0.460214 |
| 5.243321 | -9.93331 | 0.466062 |
| 6.743284 | -10.9135 | 0.454347 |
| 5.396744 | -11.8612 | 0.452726 |
| 6.858546 | -12.4633 | 0.442517 |
| 8.392068 | -11.364 | 0.443406 |
| 9.626885 | -10.7568 | 0.440606 |
| 9.817762 | -12.6846 | 0.424744 |
| 8.395041 | -12.9519 | 0.430995 |
| 7.23605 | -14.0505 | 0.427218 |
| 5.474765 | -13.5246 | 0.43917 |
| 4.163908 | -13.527 | 0.443168 |
| 3.829663 | -15.0399 | 0.430135 |
| 5.739698 | -14.9985 | 0.424541 |
| 4.730731 | -16.2103 | 0.415434 |
| 5.145122 | -17.4949 | 0.399102 |
| 6.605154 | -17.1519 | 0.397244 |
| 5.747132 | -18.9683 | 0.377309 |
| 6.161664 | -20.3285 | 0.354926 |
| 5.489916 | -21.6153 | 0.336185 |
| 6.502565 | -22.3695 | 0.316701 |
| 7.777455 | -23.161 | 0.291673 |
| 9.461781 | -22.5907 | 0.290252 |
| 8.186536 | -21.6102 | 0.320515 |
| 7.622616 | -20.477 | 0.344411 |
| 9.194936 | -20.096 | 0.339906 |
| 8.480355 | -18.5094 | 0.369153 |
| 7.28214 | -18.6629 | 0.374131 |
| 8.102709 | -16.8466 | 0.393108 |
| 6.976923 | -15.6767 | 0.412286 |
| 8.548252 | -14.7664 | 0.413414 |
| 9.29972 | -16.0504 | 0.395018 |
| 9.864206 | -17.486 | 0.373482 |
| 9.895641 | -14.2723 | 0.409832 |
| -9.59297 | -7.35266 | 0.460023 |
| -8.01867 | -8.03021 | 0.465272 |
| -8.87818 | -9.05263 | 0.45552 |
| -6.74293 | -9.27544 | 0.464451 |
| -5.39575 | -8.66797 | 0.472615 |
| -4.19853 | -7.98518 | 0.479355 |
| -6.48409 | -7.49801 | 0.474389 |
| -7.83431 | -6.47977 | 0.472877 |
| -8.8849 | -5.46096 | 0.471272 |
| -7.12504 | -5.23079 | 0.480309 |
| -6.1526 | -4.51062 | 0.486097 |
| -4.61674 | -4.65894 | 0.49046 |
| -5.6621 | -6.43785 | 0.481658 |
| -4.42643 | -6.28429 | 0.485841 |
| -3.14864 | -8.62592 | 0.478496 |
| -1.98519 | -9.90916 | 0.473243 |
| -1.42559 | -8.73608 | 0.480244 |
| -1.46616 | -7.07264 | 0.487834 |
| -2.73984 | -6.92382 | 0.486892 |
| -2.96718 | -5.52539 | 0.491514 |
| -2.44532 | -4.20115 | 0.495978 |
| -3.94464 | -3.56126 | 0.494671 |
| -5.85581 | -2.99777 | 0.490387 |
| -7.57808 | -3.30348 | 0.48316 |
| -8.66309 | -3.91045 | 0.476727 |
| -9.48982 | -2.43753 | 0.475058 |
| -9.71765 | -0.77444 | 0.475344 |
| -9.72126 | 1.153717 | 0.475107 |
| -8.63845 | 2.932701 | 0.478818 |
| -7.36646 | 3.69125 | 0.483291 |
| -9.01567 | 4.368661 | 0.473766 |
| -8.45628 | 5.655163 | 0.47284 |
| -7.22154 | 6.300219 | 0.476284 |
| -6.43715 | 7.435917 | 0.474842 |
| -8.90898 | 7.393438 | 0.463686 |
| -7.8993 | 8.227104 | 0.464879 |
| -6.10283 | 8.91103 | 0.468917 |
| -9.17497 | 9.434523 | 0.451678 |
| -7.56506 | 9.740023 | 0.458185 |
| -4.98094 | 9.820523 | 0.467538 |
| -3.70753 | 9.82293 | 0.470833 |
| -4.75275 | 7.968402 | 0.477978 |
| -3.1805 | 8.387253 | 0.479627 |
| -3.10312 | 7.064148 | 0.485712 |
| -4.97478 | 6.531309 | 0.483432 |
| -5.01004 | 5.35922 | 0.48736 |
| -6.61988 | 5.015912 | 0.482974 |
| -5.79336 | 3.656416 | 0.489339 |
| -6.01553 | 2.294937 | 0.490908 |
| -7.47521 | 1.762878 | 0.48587 |
| -8.37217 | 0.740388 | 0.482492 |
| -8.21953 | -0.77161 | 0.483214 |
| -7.61816 | -1.90469 | 0.485096 |
| -5.48326 | -1.93846 | 0.493085 |
| -6.3844 | -0.73033 | 0.490954 |
| -6.4991 | 0.517086 | 0.490607 |
| -4.81562 | 1.541062 | 0.49543 |
| -4.73802 | 0.104536 | 0.496309 |
| -3.98705 | -0.91484 | 0.497926 |
| -3.68495 | -2.23752 | 0.497403 |
| -2.11178 | -2.31016 | 0.499877 |
| -1.21107 | -3.29144 | 0.499174 |
| -1.20753 | -5.1818 | 0.494652 |
| -0.19537 | -5.67138 | 0.493546 |
| 0.967372 | -6.57655 | 0.490126 |
| -0.1541 | -7.71289 | 0.485718 |
| -0.00039 | -9.79199 | 0.475092 |
| 0.971337 | -8.69375 | 0.480777 |
| 2.131178 | -8.04883 | 0.482874 |
| 2.808448 | -9.71107 | 0.473225 |
| 4.156333 | -9.48168 | 0.471744 |
| 4.640249 | -7.89286 | 0.47864 |
| 3.36635 | -7.63062 | 0.482806 |
| 2.127425 | -6.04506 | 0.491018 |
| 1.300555 | -4.49653 | 0.496464 |
| 0.250454 | -3.74237 | 0.498676 |
| 0.322741 | -2.34336 | 0.501055 |
| -0.24146 | -1.05899 | 0.50229 |
| -1.73973 | -0.98621 | 0.501502 |
| -2.64058 | 0.070694 | 0.500668 |
| -3.20479 | 1.355071 | 0.499228 |
| -4.06925 | 2.979146 | 0.495467 |
| -4.03413 | 4.226849 | 0.492995 |
| -2.42422 | 4.53235 | 0.495188 |
| -0.77742 | 5.140378 | 0.495015 |
| -3.02573 | 5.741043 | 0.490719 |
| -1.41653 | 6.424614 | 0.490385 |
| -0.89438 | 7.597624 | 0.486004 |
| -1.64549 | 8.692612 | 0.480268 |
| 0.07657 | 9.111747 | 0.478874 |
| 1.873325 | 9.644444 | 0.474903 |
| 1.389338 | 8.093434 | 0.483424 |
| 0.679642 | 7.071299 | 0.488325 |
| 0.607355 | 5.672296 | 0.493449 |
| 0.086411 | 3.856567 | 0.498448 |
| -1.41093 | 3.437857 | 0.498749 |
| -2.42054 | 2.566384 | 0.499133 |
| -1.29397 | 0.980611 | 0.501883 |
| -0.47177 | 1.927345 | 0.501519 |
| 0.875403 | 2.534807 | 0.500608 |
| 0.055826 | 0.189212 | 0.502609 |
| 1.439748 | 1.174815 | 0.501655 |
| 2.673928 | 2.122328 | 0.499355 |
| 1.741921 | -0.18567 | 0.501762 |
| 1.669918 | -1.7359 | 0.500997 |
| 2.570207 | -2.49035 | 0.499031 |
| 2.423014 | -3.88949 | 0.496722 |
| 3.772528 | -4.52966 | 0.492821 |
| 3.475523 | -5.92909 | 0.489253 |
| 4.973999 | -6.11529 | 0.484959 |
| 6.02587 | -7.81463 | 0.47465 |
| 6.552974 | -9.28811 | 0.465143 |
| 7.977252 | -9.85252 | 0.45554 |
| 8.536287 | -8.37699 | 0.460951 |
| 7.261184 | -7.47203 | 0.471334 |
| 8.270504 | -6.44932 | 0.470905 |
| 6.059712 | -5.8864 | 0.48228 |
| 7.40696 | -5.31674 | 0.478845 |
| 9.656267 | -6.4467 | 0.463452 |
| 9.586034 | -8.94211 | 0.452093 |
| 9.80254 | -4.55607 | 0.468858 |
| 8.641212 | -4.40704 | 0.475621 |
| 6.393392 | -4.07102 | 0.486314 |
| 5.082252 | -3.92227 | 0.490977 |
| 4.218637 | -2.75188 | 0.495485 |
| 3.916393 | -1.35358 | 0.497802 |
| 3.090018 | -0.0697 | 0.499945 |
| 4.400167 | 0.310846 | 0.497157 |
| 3.760633 | 1.821925 | 0.49772 |
| 3.008032 | 3.710861 | 0.496207 |
| 1.659156 | 4.010769 | 0.497332 |
| 2.068308 | 5.523829 | 0.49281 |
| 2.702885 | 6.659244 | 0.487984 |
| 3.037131 | 8.172164 | 0.480928 |
| 4.122349 | 8.665709 | 0.476212 |
| 5.243321 | 10.06669 | 0.465258 |
| 6.743284 | 9.086542 | 0.465497 |
| 5.396744 | 8.138817 | 0.475245 |
| 6.858546 | 7.536665 | 0.472736 |
| 8.392068 | 8.635973 | 0.460352 |
| 9.626885 | 9.243222 | 0.450145 |
| 9.817762 | 7.315416 | 0.458857 |
| 8.395041 | 7.048077 | 0.467868 |
| 7.23605 | 5.949477 | 0.477487 |
| 5.474765 | 6.475448 | 0.482124 |
| 4.163908 | 6.47297 | 0.485797 |
| 3.829663 | 4.960051 | 0.491533 |
| 5.739698 | 5.001469 | 0.486167 |
| 4.730731 | 3.789732 | 0.492254 |
| 5.145122 | 2.505071 | 0.493391 |
| 6.605154 | 2.848096 | 0.487959 |
| 5.747132 | 1.031729 | 0.493008 |
| 6.161664 | -0.32855 | 0.491874 |
| 5.489916 | -1.61526 | 0.493391 |
| 6.502565 | -2.36949 | 0.489063 |
| 7.777455 | -3.16103 | 0.482538 |
| 9.461781 | -2.59074 | 0.474988 |
| 8.186536 | -1.61016 | 0.482791 |
| 7.622616 | -0.47701 | 0.486059 |
| 9.194936 | -0.09597 | 0.478427 |
| 8.480355 | 1.490582 | 0.481473 |
| 7.28214 | 1.337088 | 0.487073 |
| 8.102709 | 3.153384 | 0.481048 |
| 6.976923 | 4.323279 | 0.483443 |
| 8.548252 | 5.233621 | 0.473737 |
| 9.29972 | 3.949598 | 0.473257 |
| 9.864206 | 2.513992 | 0.472801 |
| 9.895641 | 5.727662 | 0.464684 |
| -9.59297 | 12.64734 | 0.426494 |
| -8.01867 | 11.96979 | 0.440864 |
| -8.87818 | 10.94737 | 0.443691 |
| -6.74293 | 10.72456 | 0.455609 |
| -5.39575 | 11.33203 | 0.456518 |
| -4.19853 | 12.01482 | 0.455147 |
| -6.48409 | 12.50199 | 0.443793 |
| -7.83431 | 13.52023 | 0.429055 |
| -8.8849 | 14.53904 | 0.413683 |
| -7.12504 | 14.76921 | 0.42089 |
| -6.1526 | 15.48939 | 0.417919 |
| -4.61674 | 15.34106 | 0.42495 |
| -5.6621 | 13.56215 | 0.438177 |
| -4.42643 | 13.71571 | 0.44082 |
| -3.14864 | 11.37408 | 0.462094 |
| -1.98519 | 10.09084 | 0.472164 |
| -1.42559 | 11.26392 | 0.465234 |
| -1.46616 | 12.92736 | 0.452896 |
| -2.73984 | 13.07618 | 0.450027 |
| -2.96718 | 14.47461 | 0.437431 |
| -2.44532 | 15.79885 | 0.425306 |
| -3.94464 | 16.43874 | 0.415218 |
| -5.85581 | 17.00223 | 0.402317 |
| -7.57808 | 16.69652 | 0.397815 |
| -8.66309 | 16.08956 | 0.398617 |
| -9.48982 | 17.56247 | 0.375193 |
| -9.71765 | 19.22556 | 0.349812 |
| -9.72126 | 21.15372 | 0.317008 |
| -8.63845 | 22.9327 | 0.289931 |
| -7.36646 | 23.69125 | 0.282572 |
| -9.01567 | 24.36866 | 0.251246 |
| -8.45628 | 25.65516 | 0.218641 |
| -7.22154 | 26.30022 | 0.209357 |
| -6.43715 | 27.43592 | 0.172358 |
| -8.90898 | 27.39344 | 0.140404 |
| -7.8993 | 28.2271 | 0.107057 |
| -6.10283 | 28.91103 | 0.086908 |
| -4.75275 | 27.9684 | 0.163452 |
| -3.1805 | 28.38725 | 0.153597 |
| -3.10312 | 27.06415 | 0.21053 |
| -4.97478 | 26.53131 | 0.219305 |
| -5.01004 | 25.35922 | 0.255085 |
| -6.61988 | 25.01591 | 0.254298 |
| -5.79336 | 23.65642 | 0.293463 |
| -6.01553 | 22.29494 | 0.320851 |
| -7.47521 | 21.76288 | 0.322487 |
| -8.37217 | 20.74039 | 0.334972 |
| -8.21953 | 19.22839 | 0.36039 |
| -7.61816 | 18.09531 | 0.380033 |
| -5.48326 | 18.06154 | 0.390665 |
| -6.3844 | 19.26967 | 0.370077 |
| -6.4991 | 20.51709 | 0.350159 |
| -4.81562 | 21.54106 | 0.340396 |
| -4.73802 | 20.10454 | 0.364514 |
| -3.98705 | 19.08516 | 0.381999 |
| -3.68495 | 17.76248 | 0.400317 |
| -2.11178 | 17.68984 | 0.404397 |
| -1.21107 | 16.70856 | 0.416955 |
| -1.20753 | 14.8182 | 0.436558 |
| -0.19537 | 14.32862 | 0.441573 |
| 0.967372 | 13.42345 | 0.449206 |
| -0.1541 | 12.28711 | 0.458522 |
| -0.00039 | 10.20801 | 0.472628 |
| 0.971337 | 11.30626 | 0.465274 |
| 2.131178 | 11.95117 | 0.45963 |
| 2.808448 | 10.28893 | 0.469785 |
| 4.156333 | 10.51833 | 0.465535 |
| 4.640249 | 12.10714 | 0.453252 |
| 3.36635 | 12.36938 | 0.454422 |
| 2.127425 | 13.95494 | 0.443501 |
| 1.300555 | 15.50347 | 0.429749 |
| 0.250454 | 16.25763 | 0.422396 |
| 0.322741 | 17.65664 | 0.406311 |
| -0.24146 | 18.94101 | 0.389753 |
| -1.73973 | 19.0138 | 0.387684 |
| -2.64058 | 20.07069 | 0.370939 |
| -3.20479 | 21.35507 | 0.348906 |
| -4.06925 | 22.97915 | 0.315847 |
| -4.03413 | 24.22685 | 0.288629 |
| -2.42422 | 24.53235 | 0.286433 |
| -0.77742 | 25.14038 | 0.273946 |
| -3.02573 | 25.74104 | 0.253115 |
| -1.41653 | 26.42461 | 0.236779 |
| -0.89438 | 27.59762 | 0.196509 |
| -1.64549 | 28.69261 | 0.144149 |
| 0.07657 | 29.11175 | 0.121395 |
| 1.873325 | 29.64444 | 0.070482 |
| 1.389338 | 28.09343 | 0.174779 |
| 0.679642 | 27.0713 | 0.216304 |
| 0.607355 | 25.6723 | 0.259857 |
| 0.086411 | 23.85657 | 0.304746 |
| -1.41093 | 23.43786 | 0.312845 |
| -2.42054 | 22.56638 | 0.328694 |
| -1.29397 | 20.98061 | 0.358606 |
| -0.47177 | 21.92735 | 0.342933 |
| 0.875403 | 22.53481 | 0.331465 |
| 0.055826 | 20.18921 | 0.37177 |
| 1.439748 | 21.17482 | 0.35523 |
| 2.673928 | 22.12233 | 0.336523 |
| 1.741921 | 19.81433 | 0.37626 |
| 1.669918 | 18.2641 | 0.397756 |
| 2.570207 | 17.50966 | 0.40585 |
| 2.423014 | 16.11051 | 0.422048 |
| 3.772528 | 15.47034 | 0.425972 |
| 3.475523 | 14.07091 | 0.440069 |
| 4.973999 | 13.88471 | 0.437685 |
| 6.02587 | 12.18538 | 0.448058 |
| 6.552974 | 10.71189 | 0.45647 |
| 7.977252 | 10.14748 | 0.453719 |
| 8.536287 | 11.62301 | 0.440741 |
| 7.261184 | 12.52798 | 0.440195 |
| 8.270504 | 13.55068 | 0.42648 |
| 6.059712 | 14.11361 | 0.431748 |
| 7.40696 | 14.68326 | 0.420368 |
| 9.656267 | 13.5533 | 0.418201 |
| 9.586034 | 11.05789 | 0.43876 |
| 9.80254 | 15.44393 | 0.398377 |
| 8.641212 | 15.59297 | 0.40425 |
| 6.393392 | 15.92898 | 0.412227 |
| 5.082252 | 16.07773 | 0.415714 |
| 4.218637 | 17.24812 | 0.405123 |
| 3.916393 | 18.64642 | 0.388235 |
| 3.090018 | 19.9303 | 0.372088 |
| 4.400167 | 20.31085 | 0.362486 |
| 3.760633 | 21.82193 | 0.339102 |
| 3.008032 | 23.71086 | 0.303773 |
| 1.659156 | 24.01077 | 0.300046 |
| 2.068308 | 25.52383 | 0.261843 |
| 2.702885 | 26.65924 | 0.226011 |
| 3.037131 | 28.17216 | 0.165097 |
| 4.122349 | 28.66571 | 0.131157 |
| 6.743284 | 29.08654 | 0.048849 |
| 5.396744 | 28.13882 | 0.14899 |
| 6.858546 | 27.53667 | 0.163033 |
| 8.392068 | 28.63597 | 0.051787 |
| 9.817762 | 27.31542 | 0.127021 |
| 8.395041 | 27.04808 | 0.165793 |
| 7.23605 | 25.94948 | 0.221168 |
| 5.474765 | 26.47545 | 0.217852 |
| 4.163908 | 26.47297 | 0.225927 |
| 3.829663 | 24.96005 | 0.271358 |
| 5.739698 | 25.00147 | 0.260622 |
| 4.730731 | 23.78973 | 0.295778 |
| 5.145122 | 22.50507 | 0.320982 |
| 6.605154 | 22.8481 | 0.306345 |
| 5.747132 | 21.03173 | 0.345245 |
| 6.161664 | 19.67145 | 0.365171 |
| 5.489916 | 18.38474 | 0.386383 |
| 6.502565 | 17.63051 | 0.391801 |
| 7.777455 | 16.83897 | 0.395039 |
| 9.461781 | 17.40926 | 0.37739 |
| 8.186536 | 18.38984 | 0.372676 |
| 7.622616 | 19.52299 | 0.359626 |
| 9.194936 | 19.90403 | 0.343062 |
| 8.480355 | 21.49058 | 0.320627 |
| 7.28214 | 21.33709 | 0.331585 |
| 8.102709 | 23.15338 | 0.289348 |
| 6.976923 | 24.32328 | 0.269995 |
| 8.548252 | 25.23362 | 0.231054 |
| 9.29972 | 23.9496 | 0.259515 |
| 9.864206 | 22.51399 | 0.288158 |
| 9.895641 | 25.72766 | 0.198352 |
| 10.40703 | -27.3527 | 0.110533 |
| 12.16569 | -26.4798 | 0.119434 |
| 11.1151 | -25.461 | 0.189694 |
| 12.87496 | -25.2308 | 0.165553 |
| 13.8474 | -24.5106 | 0.173692 |
| 15.38326 | -24.6589 | 0.12458 |
| 17.55468 | -24.2011 | 0.041506 |
| 16.05536 | -23.5613 | 0.156354 |
| 14.14419 | -22.9978 | 0.219116 |
| 12.42192 | -23.3035 | 0.238498 |
| 11.33691 | -23.9104 | 0.236798 |
| 10.51018 | -22.4375 | 0.283382 |
| 10.28235 | -20.7744 | 0.319073 |
| 10.27874 | -18.8463 | 0.351101 |
| 11.36155 | -17.0673 | 0.366917 |
| 12.63354 | -16.3088 | 0.364921 |
| 10.98433 | -15.6313 | 0.387523 |
| 11.54372 | -14.3448 | 0.396815 |
| 12.77846 | -13.6998 | 0.392568 |
| 13.56285 | -12.5641 | 0.395832 |
| 11.09102 | -12.6066 | 0.416519 |
| 12.1007 | -11.7729 | 0.415476 |
| 13.89717 | -11.089 | 0.404845 |
| 10.82503 | -10.5655 | 0.434051 |
| 12.43494 | -10.26 | 0.423881 |
| 15.01906 | -10.1795 | 0.400279 |
| 16.29247 | -10.1771 | 0.386064 |
| 15.24725 | -12.0316 | 0.383063 |
| 16.8195 | -11.6127 | 0.367921 |
| 16.89688 | -12.9359 | 0.354282 |
| 15.02522 | -13.4687 | 0.371938 |
| 14.98996 | -14.6408 | 0.359704 |
| 13.38012 | -14.9841 | 0.373298 |
| 14.20664 | -16.3436 | 0.347846 |
| 13.98447 | -17.7051 | 0.331274 |
| 12.52479 | -18.2371 | 0.339465 |
| 11.62783 | -19.2596 | 0.332499 |
| 11.78047 | -20.7716 | 0.304241 |
| 12.38184 | -21.9047 | 0.273698 |
| 14.51674 | -21.9385 | 0.241611 |
| 13.6156 | -20.7303 | 0.282775 |
| 13.5009 | -19.4829 | 0.308083 |
| 15.18438 | -18.4589 | 0.303752 |
| 15.26198 | -19.8955 | 0.275929 |
| 16.01295 | -20.9148 | 0.240556 |
| 16.31505 | -22.2375 | 0.197748 |
| 17.88822 | -22.3102 | 0.151962 |
| 18.78894 | -23.2914 | 0.03548 |
| 19.75854 | -21.059 | 0.136233 |
| 18.26027 | -20.9862 | 0.188169 |
| 17.35942 | -19.9293 | 0.237808 |
| 16.79522 | -18.6449 | 0.275443 |
| 15.93075 | -17.0209 | 0.316337 |
| 15.96587 | -15.7732 | 0.333526 |
| 17.57578 | -15.4677 | 0.314263 |
| 19.22258 | -14.8596 | 0.294834 |
| 16.97427 | -14.259 | 0.338645 |
| 18.58348 | -13.5754 | 0.322428 |
| 19.10562 | -12.4024 | 0.327092 |
| 18.35451 | -11.3074 | 0.349535 |
| 20.07657 | -10.8883 | 0.325898 |
| 21.87333 | -10.3556 | 0.297033 |
| 21.38934 | -11.9066 | 0.290541 |
| 20.67964 | -12.9287 | 0.292692 |
| 20.60736 | -14.3277 | 0.275324 |
| 20.08641 | -16.1434 | 0.257339 |
| 18.58907 | -16.5621 | 0.280419 |
| 17.57946 | -17.4336 | 0.283846 |
| 18.70603 | -19.0194 | 0.229931 |
| 19.52823 | -18.0727 | 0.232167 |
| 20.8754 | -17.4652 | 0.211382 |
| 20.05583 | -19.8108 | 0.17192 |
| 21.43975 | -18.8252 | 0.155325 |
| 22.67393 | -17.8777 | 0.1364 |
| 21.74192 | -20.1857 | 0.074602 |
| 23.76063 | -18.1781 | 0.037425 |
| 23.00803 | -16.2891 | 0.171918 |
| 21.65916 | -15.9892 | 0.221777 |
| 22.06831 | -14.4762 | 0.238961 |
| 22.70289 | -13.3408 | 0.240817 |
| 23.03713 | -11.8278 | 0.253751 |
| 24.12235 | -11.3343 | 0.230723 |
| 25.24332 | -9.93331 | 0.214624 |
| 26.74328 | -10.9135 | 0.135793 |
| 25.39674 | -11.8612 | 0.179133 |
| 26.85855 | -12.4633 | 0.080844 |
| 25.47477 | -13.5246 | 0.138282 |
| 24.16391 | -13.527 | 0.193307 |
| 23.82966 | -15.0399 | 0.17245 |
| 25.7397 | -14.9985 | 0.059263 |
| 24.73073 | -16.2103 | 0.084799 |
| 10.40703 | -7.35266 | 0.455028 |
| 11.98133 | -8.03021 | 0.440717 |
| 11.12182 | -9.05263 | 0.441478 |
| 13.25707 | -9.27544 | 0.423255 |
| 14.60425 | -8.66797 | 0.41433 |
| 15.80147 | -7.98518 | 0.405762 |
| 13.51591 | -7.49801 | 0.430777 |
| 12.16569 | -6.47977 | 0.446427 |
| 11.1151 | -5.46096 | 0.457796 |
| 12.87496 | -5.23079 | 0.44544 |
| 13.8474 | -4.51062 | 0.439423 |
| 15.38326 | -4.65894 | 0.424392 |
| 14.3379 | -6.43785 | 0.428122 |
| 15.57357 | -6.28429 | 0.416488 |
| 16.85136 | -8.62592 | 0.389913 |
| 18.01481 | -9.90916 | 0.366018 |
| 18.57441 | -8.73608 | 0.366554 |
| 18.53384 | -7.07264 | 0.377049 |
| 17.26016 | -6.92382 | 0.394395 |
| 17.03282 | -5.52539 | 0.403265 |
| 17.55468 | -4.20115 | 0.401461 |
| 16.05536 | -3.56126 | 0.420369 |
| 14.14419 | -2.99777 | 0.440396 |
| 12.42192 | -3.30348 | 0.454149 |
| 11.33691 | -3.91045 | 0.460715 |
| 10.51018 | -2.43753 | 0.468991 |
| 10.28235 | -0.77444 | 0.471997 |
| 10.27874 | 1.153717 | 0.471802 |
| 11.36155 | 2.932701 | 0.462579 |
| 12.63354 | 3.69125 | 0.451665 |
| 10.98433 | 4.368661 | 0.461955 |
| 11.54372 | 5.655163 | 0.454142 |
| 12.77846 | 6.300219 | 0.442324 |
| 13.56285 | 7.435917 | 0.430665 |
| 11.09102 | 7.393438 | 0.450283 |
| 12.1007 | 8.227104 | 0.438778 |
| 13.89717 | 8.91103 | 0.419674 |
| 10.82503 | 9.434523 | 0.441304 |
| 12.43494 | 9.740023 | 0.42731 |
| 15.01906 | 9.820523 | 0.402788 |
| 16.29247 | 9.82293 | 0.38863 |
| 15.24725 | 7.968402 | 0.411762 |
| 16.8195 | 8.387253 | 0.391757 |
| 16.89688 | 7.064148 | 0.398095 |
| 15.02522 | 6.531309 | 0.421051 |
| 14.98996 | 5.35922 | 0.42602 |
| 13.38012 | 5.015912 | 0.441942 |
| 14.20664 | 3.656416 | 0.438431 |
| 13.98447 | 2.294937 | 0.443006 |
| 12.52479 | 1.762878 | 0.455765 |
| 11.62783 | 0.740388 | 0.463164 |
| 11.78047 | -0.77161 | 0.462066 |
| 12.38184 | -1.90469 | 0.4567 |
| 14.51674 | -1.93846 | 0.438656 |
| 13.6156 | -0.73033 | 0.447706 |
| 13.5009 | 0.517086 | 0.448763 |
| 15.18438 | 1.541062 | 0.432714 |
| 15.26198 | 0.104536 | 0.432714 |
| 16.01295 | -0.91484 | 0.424756 |
| 16.31505 | -2.23752 | 0.420126 |
| 17.88822 | -2.31016 | 0.401633 |
| 18.78894 | -3.29144 | 0.387935 |
| 18.79248 | -5.1818 | 0.382047 |
| 19.80463 | -5.67138 | 0.36538 |
| 20.96737 | -6.57655 | 0.342175 |
| 19.8459 | -7.71289 | 0.354081 |
| 19.99961 | -9.79199 | 0.336806 |
| 20.97134 | -8.69375 | 0.328576 |
| 22.13118 | -8.04883 | 0.311384 |
| 22.80845 | -9.71107 | 0.283073 |
| 24.15633 | -9.48168 | 0.252185 |
| 24.64025 | -7.89286 | 0.254397 |
| 23.36635 | -7.63062 | 0.288143 |
| 22.12743 | -6.04506 | 0.323935 |
| 21.30056 | -4.49653 | 0.345826 |
| 20.25045 | -3.74237 | 0.365496 |
| 20.32274 | -2.34336 | 0.367633 |
| 19.75854 | -1.05899 | 0.377794 |
| 18.26027 | -0.98621 | 0.398446 |
| 17.35942 | 0.070694 | 0.409924 |
| 16.79522 | 1.355071 | 0.415852 |
| 15.93075 | 2.979146 | 0.422963 |
| 15.96587 | 4.226849 | 0.419595 |
| 17.57578 | 4.53235 | 0.400189 |
| 19.22258 | 5.140378 | 0.376152 |
| 16.97427 | 5.741043 | 0.403112 |
| 18.58348 | 6.424614 | 0.37961 |
| 19.10562 | 7.597624 | 0.366009 |
| 18.35451 | 8.692612 | 0.369937 |
| 20.07657 | 9.111747 | 0.340855 |
| 21.87333 | 9.644444 | 0.303679 |
| 21.38934 | 8.093434 | 0.325301 |
| 20.67964 | 7.071299 | 0.344314 |
| 20.60736 | 5.672296 | 0.352695 |
| 20.08641 | 3.856567 | 0.367697 |
| 18.58907 | 3.437857 | 0.390274 |
| 17.57946 | 2.566384 | 0.405009 |
| 18.70603 | 0.980611 | 0.392603 |
| 19.52823 | 1.927345 | 0.380184 |
| 20.8754 | 2.534807 | 0.35847 |
| 20.05583 | 0.189212 | 0.373779 |
| 21.43975 | 1.174815 | 0.351018 |
| 22.67393 | 2.122328 | 0.327192 |
| 21.74192 | -0.18567 | 0.346307 |
| 21.66992 | -1.7359 | 0.346367 |
| 22.57021 | -2.49035 | 0.328474 |
| 22.42301 | -3.88949 | 0.327488 |
| 23.77253 | -4.52966 | 0.297047 |
| 23.47552 | -5.92909 | 0.296761 |
| 24.974 | -6.11529 | 0.258958 |
| 26.02587 | -7.81463 | 0.212971 |
| 26.55297 | -9.28811 | 0.174652 |
| 27.97725 | -9.85252 | 0.075302 |
| 28.53629 | -8.37699 | 0.065974 |
| 27.26118 | -7.47203 | 0.168373 |
| 28.2705 | -6.44932 | 0.128891 |
| 26.05971 | -5.8864 | 0.228643 |
| 27.40696 | -5.31674 | 0.183982 |
| 28.64121 | -4.40704 | 0.130056 |
| 26.39339 | -4.07102 | 0.228995 |
| 25.08225 | -3.92227 | 0.2678 |
| 24.21864 | -2.75188 | 0.29302 |
| 23.91639 | -1.35358 | 0.302582 |
| 23.09002 | -0.0697 | 0.320893 |
| 24.40017 | 0.310846 | 0.292373 |
| 23.76063 | 1.821925 | 0.305324 |
| 23.00803 | 3.710861 | 0.316495 |
| 21.65916 | 4.010769 | 0.341221 |
| 22.06831 | 5.523829 | 0.327658 |
| 22.70289 | 6.659244 | 0.309035 |
| 23.03713 | 8.172164 | 0.291397 |
| 24.12235 | 8.665709 | 0.261179 |
| 25.24332 | 10.06669 | 0.212872 |
| 26.74328 | 9.086542 | 0.169399 |
| 25.39674 | 8.138817 | 0.230186 |
| 26.85855 | 7.536665 | 0.184915 |
| 28.39207 | 8.635973 | 0.073623 |
| 28.39504 | 7.048077 | 0.111192 |
| 27.23605 | 5.949477 | 0.185661 |
| 25.47477 | 6.475448 | 0.242271 |
| 24.16391 | 6.47297 | 0.277432 |
| 23.82966 | 4.960051 | 0.293814 |
| 25.7397 | 5.001469 | 0.244203 |
| 24.73073 | 3.789732 | 0.277343 |
| 25.14512 | 2.505071 | 0.270901 |
| 26.60515 | 2.848096 | 0.227296 |
| 25.74713 | 1.031729 | 0.257391 |
| 26.16166 | -0.32855 | 0.245924 |
| 25.48992 | -1.61526 | 0.263659 |
| 26.50257 | -2.36949 | 0.23215 |
| 27.77746 | -3.16103 | 0.182319 |
| 29.46178 | -2.59074 | 0.084257 |
| 28.18654 | -1.61016 | 0.169974 |
| 27.62262 | -0.47701 | 0.195931 |
| 29.19494 | -0.09597 | 0.115647 |
| 28.48036 | 1.490582 | 0.155954 |
| 27.28214 | 1.337088 | 0.207842 |
| 28.10271 | 3.153384 | 0.167786 |
| 26.97692 | 4.323279 | 0.207611 |
| 28.54825 | 5.233621 | 0.127161 |
| 29.29972 | 3.949598 | 0.085309 |
| 29.86421 | 2.513992 | 0.022534 |
| 10.40703 | 12.64734 | 0.421102 |
| 11.98133 | 11.96979 | 0.414868 |
| 11.12182 | 10.94737 | 0.429262 |
| 13.25707 | 10.72456 | 0.413533 |
| 14.60425 | 11.33203 | 0.39587 |
| 15.80147 | 12.01482 | 0.376856 |
| 13.51591 | 12.50199 | 0.396833 |
| 12.16569 | 13.52023 | 0.399715 |
| 11.1151 | 14.53904 | 0.398263 |
| 12.87496 | 14.76921 | 0.380617 |
| 13.8474 | 15.48939 | 0.362572 |
| 15.38326 | 15.34106 | 0.346612 |
| 14.3379 | 13.56215 | 0.378541 |
| 15.57357 | 13.71571 | 0.362964 |
| 16.85136 | 11.37408 | 0.3696 |
| 18.01481 | 10.09084 | 0.364622 |
| 18.57441 | 11.26392 | 0.346656 |
| 18.53384 | 12.92736 | 0.330602 |
| 17.26016 | 13.07618 | 0.347863 |
| 17.03282 | 14.47461 | 0.335234 |
| 17.55468 | 15.79885 | 0.30994 |
| 16.05536 | 16.43874 | 0.323137 |
| 14.14419 | 17.00223 | 0.339601 |
| 12.42192 | 16.69652 | 0.362027 |
| 11.33691 | 16.08956 | 0.379322 |
| 10.51018 | 17.56247 | 0.36748 |
| 10.28235 | 19.22556 | 0.345251 |
| 10.27874 | 21.15372 | 0.312033 |
| 11.36155 | 22.9327 | 0.262245 |
| 12.63354 | 23.69125 | 0.224227 |
| 10.98433 | 24.36866 | 0.228195 |
| 11.54372 | 25.65516 | 0.17456 |
| 12.77846 | 26.30022 | 0.112401 |
| 11.09102 | 27.39344 | 0.086391 |
| 14.98996 | 25.35922 | 0.095087 |
| 13.38012 | 25.01591 | 0.163449 |
| 14.20664 | 23.65642 | 0.197204 |
| 13.98447 | 22.29494 | 0.241264 |
| 12.52479 | 21.76288 | 0.275045 |
| 11.62783 | 20.74039 | 0.306479 |
| 11.78047 | 19.22839 | 0.331496 |
| 12.38184 | 18.09531 | 0.343048 |
| 14.51674 | 18.06154 | 0.319226 |
| 13.6156 | 19.26967 | 0.310422 |
| 13.5009 | 20.51709 | 0.288624 |
| 15.18438 | 21.54106 | 0.240125 |
| 15.26198 | 20.10454 | 0.271642 |
| 16.01295 | 19.08516 | 0.280018 |
| 16.31505 | 17.76248 | 0.298914 |
| 17.88822 | 17.68984 | 0.273804 |
| 18.78894 | 16.70856 | 0.274172 |
| 18.79248 | 14.8182 | 0.303087 |
| 19.80463 | 14.32862 | 0.291379 |
| 20.96737 | 13.42345 | 0.280438 |
| 19.8459 | 12.28711 | 0.315744 |
| 19.99961 | 10.20801 | 0.333321 |
| 20.97134 | 11.30626 | 0.305443 |
| 22.13118 | 11.95117 | 0.273958 |
| 22.80845 | 10.28893 | 0.277284 |
| 24.15633 | 10.51833 | 0.24037 |
| 24.64025 | 12.10714 | 0.20263 |
| 23.36635 | 12.36938 | 0.237536 |
| 22.12743 | 13.95494 | 0.246025 |
| 21.30056 | 15.50347 | 0.240423 |
| 20.25045 | 16.25763 | 0.25165 |
| 20.32274 | 17.65664 | 0.22178 |
| 19.75854 | 18.94101 | 0.205766 |
| 18.26027 | 19.0138 | 0.239903 |
| 17.35942 | 20.07069 | 0.234447 |
| 16.79522 | 21.35507 | 0.213166 |
| 15.93075 | 22.97915 | 0.182126 |
| 15.96587 | 24.22685 | 0.12776 |
| 18.58907 | 23.43786 | 0.037886 |
| 17.57946 | 22.56638 | 0.151455 |
| 18.70603 | 20.98061 | 0.175636 |
| 19.52823 | 21.92735 | 0.103061 |
| 20.05583 | 20.18921 | 0.159083 |
| 21.74192 | 19.81433 | 0.098666 |
| 21.66992 | 18.2641 | 0.164869 |
| 22.57021 | 17.50966 | 0.153551 |
| 22.42301 | 16.11051 | 0.196572 |
| 23.77253 | 15.47034 | 0.163757 |
| 23.47552 | 14.07091 | 0.205815 |
| 24.974 | 13.88471 | 0.153108 |
| 26.02587 | 12.18538 | 0.14429 |
| 26.55297 | 10.71189 | 0.150035 |
| 27.97725 | 10.14748 | 0.06336 |
| 26.05971 | 14.11361 | 0.078041 |
| 25.08225 | 16.07773 | 0.058967 |
| 24.21864 | 17.24812 | 0.066932 |
